# Pragmatic language disorders as predictors of psychopathological trajectories in childhood and adolescence: a systematic review

**DOI:** 10.3389/frcha.2026.1860959

**Published:** 2026-07-22

**Authors:** Sergio Melogno, Paolo Stievano, Andrea Paglialunga

**Affiliations:** 1 Department of Economic, Psychological, Communication, Education and Motor Sciences, Università degli Studi Niccolò Cusano, Roma, Italy; 2 ASL ROMA2, Roma, Italy

**Keywords:** child and adolescent psychiatry, developmental cascades, longitudinal studies, pragmatic language, predictive validity, psychopathology, social (pragmatic) communication disorder, transdiagnostic

## Abstract

**Introduction:**

Pragmatic language competence, defined as the ability to use language appropriately in social contexts, is fundamental to adaptive development. Although pragmatic deficits are recognized across multiple neurodevelopmental conditions, their predictive validity for later psychopathology remains underexplored. This systematic review examines whether early pragmatic language impairments predict subsequent psychopathological outcomes in children and adolescents, thereby providing indirect, dimensional evidence relevant to the ongoing nosological debate surrounding Social (Pragmatic) Communication Disorder (SPCD), although no included study diagnosed SPCD according to Diagnostic and Statistical Manual of Mental Disorders, fifth edition (DSM-5) criteria.

**Methods:**

A systematic search was conducted for longitudinal studies published between 1980 and 2025 that assessed pragmatic language skills in children aged 4 to 14 years and measured psychopathological outcomes after a minimum six-month follow-up. Methodological quality was evaluated using the Newcastle-Ottawa Scale (NOS), and the review was conducted in accordance with the Preferred Reporting Items for Systematic Reviews and Meta-Analyses (PRISMA) 2020 guidelines.

**Results:**

Sixteen studies met the inclusion criteria, with eight rated as high quality, seven as moderate quality, and one as low quality. Findings suggest that pragmatic deficits may predict internalizing symptoms (anxiety, depression, suicidality), externalizing problems (hyperactivity, conduct difficulties), and persistent peer relationship difficulties. These associations appeared robust after controlling for structural language abilities and general cognitive functioning. Evidence for psychotic outcomes was less consistent.

**Discussion:**

Given the heterogeneity and methodological limitations of the evidence base, these findings should be regarded as convergent and suggestive rather than definitive. The results indicate that early pragmatic language impairments may serve as a transdiagnostic marker of vulnerability for diverse psychopathological trajectories, supporting dimensional models of psychopathology such as the Hierarchical Taxonomy of Psychopathology (HiTOP) and the Research Domain Criteria (RDoC), an interpretation that the present evidence supports indirectly rather than confirms. Routine screening for pragmatic difficulties in early childhood may identify at-risk individuals and inform preventive interventions in child and adolescent mental health services. Future research should address cross-cultural generalizability, employ multimodal assessment approaches, and test bidirectional relationships between pragmatic development and psychopathology.

## 1 Introduction

Pragmatic competence, the ability to use language appropriately in social contexts, is fundamental to human communication and to the development of social relationships across the lifespan (1). Early systematic efforts to measure qualitative abnormalities in language use, distinct from structural linguistic deficits, were pioneered by Rapin and Allen (2) with their description of semantic-pragmatic disorder and later by Bishop (3) with the Children's Communication Checklist (CCC). These studies identified a heterogeneous group of children whose pragmatic difficulties overlap with, but are not fully explained by, diagnoses such as Autism Spectrum Disorder (ASD) or Developmental Language Disorder (DLD).

Pragmatic competence is conventionally subdivided into several interrelated subdomains, each capturing a distinct aspect of language use in interaction. These typically include the contextual and discursive use of language, encompassing the production of relevant and coherent contributions; topic management and conversational repair; inferential comprehension, including the interpretation of presuppositions, implicatures, and non-literal language such as irony and figurative speech; the selection of speech acts appropriate to the social and communicative context; turn-taking and the regulation of communicative exchange; and the non-verbal aspects of communication such as prosody, gaze, and gesture (3, 4). The present review treats pragmatic language as a multidimensional construct that subsumes these subdomains, while recognising that included studies vary in the breadth and specificity with which they operationalise pragmatic ability.

The introduction of Social (Pragmatic) Communication Disorder (SPCD) in the Diagnostic and Statistical Manual of Mental Disorders, fifth edition (DSM-5) (5) marked a significant development in the psychiatric classification of these impairments. SPCD was designed to categorize individuals with pragmatic deficits not attributable to ASD or structural language disorders. However, this new diagnosis intensified debates about the nosological boundaries between ASD, DLD, and pragmatic impairments (4, 6). Recent reviews acknowledge that SPCD presents distinctive clinical features and has potential utility as a diagnostic category (7, 8), while at the same time documenting persistent overlap with adjacent neurodevelopmental conditions, high comorbidity, and a paucity of SPCD-specific assessment tools (7, 9). Within this active debate, some authors emphasize the categorical specificity of SPCD as supported by the DSM-5, whereas others propose that pragmatic difficulties may be more parsimoniously conceptualized along a continuum spanning DLD, SPCD, and ASD (8, 9).

Clarifying these nosological boundaries depends on the concept of diagnostic validity. Robins and Guze (10) proposed foundational criteria for establishing a diagnosis's scientific legitimacy, emphasizing the role of longitudinal and biological validators. Kendler (11–13) further argued that predictive validity, understood as a diagnosis's ability to forecast longitudinal outcomes, is a key indicator of its scientific status and should be refined through epistemic iteration based on cumulative evidence. Consequently, pragmatic language deficits should be assessed not only by concurrent linguistic measures but also by their power to predict future socio-emotional and psychopathological trajectories. It should be noted, however, that the existing longitudinal literature predominantly measures pragmatic language as a continuous variable using screening instruments [e.g., CCC, Social and Communication Disorders Checklist (SCDC)] rather than applying DSM-5 diagnostic criteria for SPCD. This review therefore examines the predictive validity of dimensionally assessed pragmatic deficits, which may inform, but cannot directly establish, the diagnostic validity of SPCD as a discrete nosological entity.

In parallel, dimensional models of psychopathology such as the Hierarchical Taxonomy of Psychopathology [HiTOP; (14)] and the Research Domain Criteria [RDoC; (15)] have reconceptualized mental disorders as dimensions rather than discrete categories. Within these frameworks, pragmatic language deficits, which are present across conditions like ASD, Attention-Deficit/Hyperactivity Disorder (ADHD), and conduct disorders, may function as transdiagnostic traits at the intersection of social cognition, language processing, and emotion regulation. A synthesis of the typical pragmatic profiles, putative underlying mechanisms, and distinctive features of these clinical conditions is provided in Supplementary Table S1 (16, 17).

The shift toward dimensional models reflects mounting empirical and conceptual concerns about the categorical organisation of psychopathology. Categorical nosologies based on polythetic, threshold-based criteria, although clinically convenient, often fail to capture the heterogeneity of symptom presentation, the high rates of within-spectrum and cross-spectrum comorbidity, and the dimensional distribution of psychopathological liability documented in epidemiological samples (13). HiTOP addresses these concerns by reorganising observed psychopathology along empirically derived spectra, including internalising, disinhibited externalising, antagonistic externalising, thought disorder, somatoform, and detachment, that span from normal-range variation to extreme manifestations, and by situating narrower syndromes within broader hierarchical levels (13).

RDoC complements this perspective from a different vantage. Rather than starting from clinical syndromes, it organises mental functioning around a small set of biobehaviourally informed domains and constructs, including systems for negative valence, positive valence, cognitive systems, social processes, arousal and regulatory systems, and sensorimotor systems (15). Within both HiTOP and RDoC, communicative competence and its constituent skills are most appropriately treated as continuous traits whose impairment incrementally raises the probability of multiple psychopathological outcomes, rather than as defining features of a single disorder. Kendler's epistemic iteration framework (11, 13) provides the broader rationale for this stance: psychiatric constructs gain validity when their predictive relations with relevant outcomes are progressively documented across populations, time points, and measurement modalities, and when their boundaries are re-evaluated in light of accumulating evidence.

The developmental cascades framework (18) provides further theoretical support, viewing development as a dynamic system in which competence or maladaptation in one area, such as language use, has cumulative effects on other domains. Pragmatic impairments may initiate negative cascades by hindering social reciprocity, promoting social withdrawal, or provoking adverse reactions from others, thereby heightening vulnerability to anxiety, depression, and behavioral disorders.

Despite this growing recognition, a significant translational gap persists between research evidence and clinical practice. Pragmatic language assessment is not routinely incorporated into developmental surveillance protocols in most countries. If pragmatic impairments can be shown to reliably predict later psychopathological outcomes, this would provide a strong rationale for incorporating standardized pragmatic assessment into early screening procedures.

Accordingly, this review synthesizes longitudinal evidence to determine whether pragmatic language disorders predict subsequent psychopathological outcomes in children and adolescents, and to evaluate the implications for clinical practice, early identification, and preventive strategies in child and adolescent psychiatry.

## 2 Methods

### 2.1 Study design and protocol

This systematic review was conducted and reported in accordance with the Preferred Reporting Items for Systematic Reviews and Meta-Analyses (PRISMA) 2020 statement. The review protocol was not prospectively registered in PROSPERO, as the registry's current eligibility criteria do not cover prognostic reviews examining the predictive validity of clinical markers. A pre-determined protocol specifying research question, eligibility criteria, search strategy, data extraction procedures, and quality assessment methods was followed; the full protocol is available from the corresponding author upon reasonable request.

### 2.2 Research question (PICOS framework)

The research question was formulated according to the PICOS (Population, Indicator/Exposure, Comparator, Outcomes, Study design) framework as follows. Population: children and adolescents aged 4 to 14 years at baseline assessment. Indicator (Exposure): pragmatic language skills, assessed through validated instruments or systematic coding schemes. Comparator: children without pragmatic language difficulties, or within-sample variation in pragmatic ability. Outcomes: quantitative measures of psychopathological symptoms, behavioral problems, or socio-emotional difficulties assessed after a minimum interval of 6 months. Study designs: longitudinal, prospective, or follow-up studies; cross-sectional studies were included only when they employed regression-based predictive analytic strategies with appropriate confounder control.

### 2.3 Participants

Eligible studies included participants aged between 4 and 14 years at the initial assessment. Studies were excluded if participants had a primary diagnosis of intellectual disability (IQ < 70). No restrictions were placed on clinical status at baseline; accordingly, the review encompassed both community-based and clinical samples, including children with developmental language disorders, autism spectrum disorder, attention-deficit/hyperactivity disorder, and traumatic brain injury, provided that pragmatic language was measured as a distinct predictor variable.

This permissive approach to clinical status reflects the dimensional framework adopted in this review (14, 15) and rests on a methodological distinction. The included studies on clinical populations (ASD, DLD, ADHD, and paediatric TBI) operationalised pragmatic competence as a graded continuous trait, measured by standardised checklists or language tasks, and modelled it as a predictor of subsequent psychopathological outcomes with statistical control for structural language and cognitive ability (19–21). Pragmatic skills were therefore not treated as categorical symptoms of the underlying clinical condition, but as quantitative individual differences within clinical groups whose predictive validity is the object of inquiry. Two of the included studies further employed pre-morbid designs that separate childhood pragmatic variation from later clinical onset: Done and Leinonen (22) assessed pragmatic ability at age 11 in a population sample where schizophrenia diagnoses emerged only in adulthood, and Sullivan et al. (23) drew on the ALSPAC birth cohort to relate childhood communication ability to adolescent psychotic experiences in the general population rather than in already-diagnosed cases. In studies on paediatric TBI, the pragmatic predictor was assessed alongside IQ and verbal working memory (21), allowing its unique contribution to be estimated net of these covariates. Consistent with this rationale, the present review evaluates the predictive validity of pragmatics as a dimensional trait, and does not adjudicate the diagnostic validity of any categorical entity, including SPCD.

### 2.4 Search strategy and data sources

A systematic search was performed across four electronic databases: Google Scholar, PubMed, ScienceDirect, and PsycINFO (APA PsycNet). The search covered articles published between January 1980 and December 2025. All database searches were conducted between November and December 2025. The search strategy was designed to be deliberately broad and inclusive, using a combination of keywords related to pragmatic language deficits, longitudinal study designs, and developmental age. This approach was adopted to account for the terminological variability that characterizes earlier research on pragmatic impairments, which employed heterogeneous labels such as “semantic-pragmatic disorder,” “pragmatic language impairment,” and various *ad hoc* measures of discourse coherence, predating the introduction of SPCD in the DSM-5 (5) and the standardization of instruments such as the CCC (3).

The primary search query used for Google Scholar and PubMed was: [“pragmatic language impairment” OR “semantic-pragmatic disorder” OR “pragmatic language difficulties” OR “social (pragmatic) communication disorder” OR “pragmatic deficits” OR “pragmatic language skills”] AND (“longitudinal” OR “follow-up” OR “prospective” OR “trajectory” OR “predict” OR “developmental outcome” OR “later adjustment” OR “future” OR “change over time”) AND (child OR adolescent OR “early childhood” OR preschool OR “school-age”). To maximize sensitivity on ScienceDirect and accommodate platform-specific limitations on Boolean operators, a broader search was conducted using only the first part of the query: [“pragmatic language impairment” OR “semantic-pragmatic disorder” OR “pragmatic language difficulties” OR “social (pragmatic) communication disorder” OR “pragmatic deficits” OR “pragmatic language skills”].

For Google Scholar, records were sorted by relevance and the first 1,000 records, corresponding to the 50 result pages that the platform makes accessible, were screened in descending order of relevance. Screening of the lower-ranked pages yielded no further potentially eligible records, indicating relevance saturation. The deliberately broad first component of the query was adopted to maximize sensitivity across the heterogeneous terminology historically used in this field, rather than to optimize precision. Full database-specific search strings, filters, search dates, and numbers of records retrieved are reported in Supplementary Table S2.

An additional search was conducted on PsycINFO (APA PsycNet) using the following strategy: Any Field: SPCD OR Any Field: pragmatic language impairment OR Any Field: semantic-pragmatic disorder OR Any Field: pragmatic language difficulties OR Any Field: social (pragmatic) communication disorder AND Any Field: pragmatic deficits AND Any Field: pragmatic language skills AND Age Group: Childhood (birth–12 yrs) OR Adolescence (13–17 yrs) AND Methodology: Longitudinal Study, filtered to Peer-Reviewed Journals only. This search yielded 40 records; however, 5 were duplicates of records already identified through the other databases, and the remaining 35 were excluded following title and abstract screening as they did not meet the eligibility criteria.

To expand the search, backward and forward citation chaining was conducted on systematic reviews focusing on broader language disorders. Despite this inclusive strategy, no studies meeting the eligibility criteria were identified prior to 2005, likely reflecting the relatively recent emergence of longitudinal research specifically targeting pragmatic language as a predictor variable.

### 2.5 Study selection and eligibility criteria

Studies were included if they met the following criteria: (1) participants were aged between 4 and 14 years at the initial assessment; (2) the study specifically investigated pragmatic language skills as a predictor variable; (3) the study employed a longitudinal, prospective, or follow-up design with a minimum duration of 6 months between baseline and outcome assessment; (4) the outcome measures included quantitative data on psychopathological symptoms or behavioral problems; and (5) the study was a peer-reviewed article published in English. Studies were excluded if they focused exclusively on social communication without a direct linguistic measure, included participants with a primary diagnosis of intellectual disability (IQ < 70), were case reports or qualitative studies, or were purely cross-sectional without employing statistical methods to examine predictive associations.

Screening of titles and abstracts and full-text assessment were performed independently by two authors. Inter-reviewer agreement was assessed using Cohen's kappa (*κ* ≈ 0.8). Disagreements were resolved by consensus; if unresolved, a third reviewer adjudicated. Cohen's kappa was computed at the level of the overall inclusion decision; the original rater-level records that would allow per-criterion concordance estimates are not retrievable, and this is acknowledged as a limitation (section 4.3).

Three studies with cross-sectional designs or short-term follow-ups (24–26) were exceptionally retained in the final synthesis. This decision was justified by their use of regression-based analytic strategies explicitly designed to test predictive relationships, their control for relevant confounders (including structural language and cognitive ability), and their reliance on large, well-characterized samples. The study by Saul et al. (26) was included because, although primarily designed to estimate prevalence, it incorporates a prospective longitudinal analysis examining whether pragmatic skills assessed at age 5 to 6 could predict academic attainment one year later, after controlling for structural language. However, findings from these studies are interpreted with appropriate caution regarding temporal precedence. To avoid conflating statistical prediction in cross-sectional data with genuine temporal prediction, the terms predict and predictive are reserved hereafter for longitudinal designs, whereas results from the three cross-sectional studies (24–26) are reported as concurrent associations. Additionally, the study by Skuse et al. (27) was classified as longitudinal because it involved temporally separated assessments: parent-reported social communication at approximately age 7 years 8 months, teacher-rated behavioral outcomes approximately one year later, and independent clinical diagnoses of autism spectrum disorder at age 11 years.

**Figure 1:**
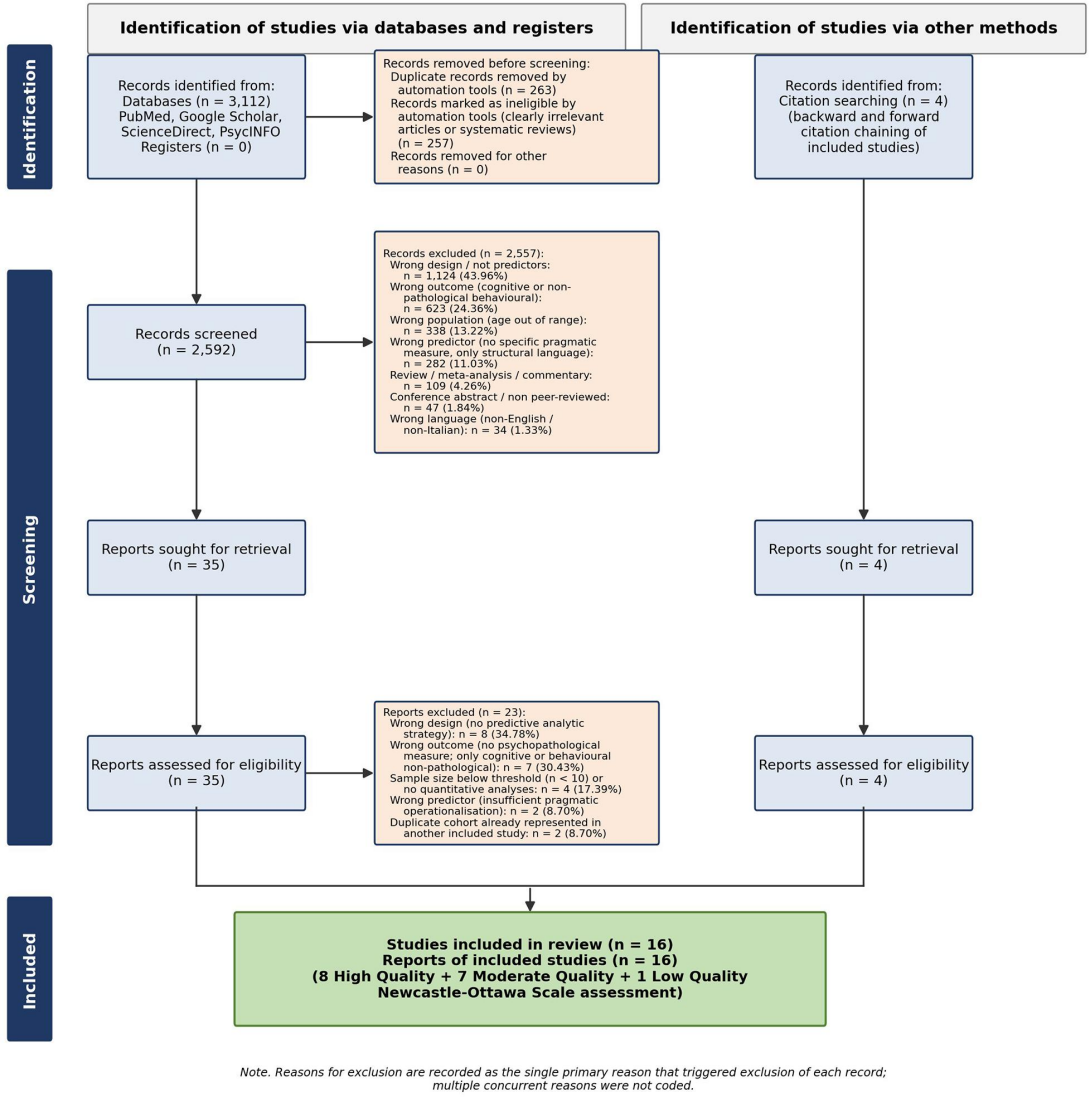
PRISMA 2020 flow diagram illustrating the systematic study selection process.

The study selection process followed a multi-stage approach comprising duplicate removal, title and abstract screening, full-text assessment against eligibility criteria, and backward and forward citation chaining. Quantitative results of each screening stage are reported in section 3.1 and illustrated in the PRISMA flow diagram (Figure 1).

### 2.6 Data extraction and synthesis

Data from the 16 included studies were systematically extracted and organized into a structured format covering: (1) general study characteristics (authors, year, country, design, follow-up duration, sample size); (2) pragmatic language measures; (3) psychopathological outcomes and measurement tools; (4) mediating or moderating factors; (5) methodological quality and predictive validity; and (6) statistical models used for predictive and trajectory analysis. Findings were synthesized narratively, organized by five psychopathological domains: (1) externalizing and behavioral problems; (2) internalizing and mood difficulties; (3) social adaptation and peer relationships; (4) mixed or broad psychopathology; and (5) severe psychopathology (psychotic experiences, schizophrenia). Within each domain, results were evaluated for strength of the predictive effect, specificity of the pragmatic effect relative to structural language and cognitive functioning, and presence of mediating or moderating variables. Given the substantial heterogeneity across included studies, a quantitative meta-analysis was not undertaken.

### 2.7 Quality assessment and risk of bias

The methodological quality and risk of bias of the included studies were assessed using the Newcastle-Ottawa Scale (NOS), adapted for longitudinal cohort studies. The NOS evaluates studies on three domains: selection of study groups (0 to 4 stars), comparability of groups (0 to 2 stars), and ascertainment of outcome and adequacy of follow-up (0 to 3 stars), for a maximum total score of 9 stars. Studies scoring 7 to 9 were classified as High Quality, 5 to 6 as Moderate Quality, and below 5 as Low Quality. Quality assessment was performed independently by two reviewers, with disagreements resolved by consensus. Results were used to weigh evidence strength during the narrative synthesis (see also Supplementary Table S3 for the full NOS scoring; pre-consensus rater-level NOS scores were not preserved in retrievable form, a limitation discussed in section 4.3). Additionally, two sensitivity analyses are reported in Supplementary Table S4 to test the robustness of the synthesis under stricter inclusion criteria than those specified in the protocol. To complement the global Newcastle-Ottawa ratings with a domain-specific appraisal, each study was additionally rated as low, moderate, or high risk across seven domains (sample selection and participation; attrition; measurement of the pragmatic exposure; measurement of the psychopathological outcome; temporal precedence of exposure relative to outcome; confounding; and shared method variance and analysis), using a scheme adapted from the QUIPS tool for prognostic-factor reviews (50) and extended with the temporal-precedence and shared-method-variance domains. These domain-level judgments are reported in Supplementary Table S5.

## 3 Results

### 3.1 Study selection

The study selection process is summarized in the PRISMA 2020 flow diagram (Figure 1). The initial database search across PubMed, Google Scholar, ScienceDirect, and PsycINFO yielded 3,112 records. Records retrieved from the four databases were processed in two spreadsheet-based steps before manual screening, each verified by hand. First, 263 duplicate records were identified by sorting and matching records on common title strings and were removed after the flagged matches had been checked manually. Second, a further 257 records were flagged as clearly ineligible by searching record titles for the term review and related strings, which identified systematic reviews and other non-empirical or manifestly out-of-scope records. Every record flagged for removal at either step was inspected by a human author before exclusion, and any record flagged in error was retained for full manual screening. After these two steps, 2,592 records remained for manual screening. Detailed abstract screening excluded 2,557 records for the reasons reported in Figure 1, after which 35 reports were sought for retrieval and assessed for eligibility at the full text stage; of these, 23 were excluded according to the criteria detailed in Figure 1. Backward and forward citation chaining of the included studies added 4 further reports, all of which met the eligibility criteria. The final synthesis therefore included 16 studies, 12 from the database search and 4 from citation chaining, comprising 13 longitudinal studies and 3 cross sectional studies with predictive analyses.

### 3.2 Study characteristics

The 16 included studies were published between 2005 and 2023. Eleven were conducted in the United Kingdom, with the remaining five from Canada, the Netherlands, Spain, Australia, and the United States. Sample sizes ranged from 23 (28) to 15,145 (22), and follow-up durations spanned from approximately 6 months (19) to over 27 years (28). A complete description of study identification variables is provided in Supplementary Table S6, while the clinical diagnostic categories of the original samples and the comparison groups used in each study, with their respective sample sizes, are reported in Supplementary Table S7. The majority of studies drew on well-established population cohorts, including the Avon Longitudinal Study of Parents and Children (ALSPAC) (23, 27, 29, 30), the National Child Development Study (NCDS) (22, 24), and the Manchester Language Study (MLS) (31–33). Clinical samples included children with developmental language disorders (28), autism spectrum disorder (19, 20), and traumatic brain injury (21).

Pragmatic language was assessed using a variety of instruments. The most frequently employed were the Children's Communication Checklist (CCC) (3) and its second edition (CCC-2), used in nine studies, followed by the Social and Communication Disorders Checklist (SCDC) in two studies. Other measures included the Comprehensive Assessment of Spoken Language (CASL) pragmatic judgment subtest, the Test of Language Competence-Expanded Edition (TLC-E), and researcher-designed coding schemes applied to free-writing tasks. The informant source varied across studies: five relied on direct assessment (including written tasks), five on parent report, four on teacher report, and two on a combination of parent and teacher reports.

Psychopathological outcomes were measured primarily through the Strengths and Difficulties Questionnaire (SDQ), employed in eight studies, alongside instruments such as the Child Behavior Checklist (CBCL), the Rutter Children's Behaviour Questionnaire, the Social Anxiety Scale for Children (SASC-R), the Moods and Feelings Questionnaire (MFQ), and the Psychosis-Like Symptom Interview (PLIKSi). A summary of the key methodological characteristics, study designs, and assessment instruments is presented in Table 1. A synthesis of the impact of pragmatic abilities on psychopathological and adaptive trajectories, including effect strengths, specificity, and intervening factors, is presented in Table 2. Complete detailed extraction data (Supplementary Tables S3, S6, S8–S12) are available in the Supplementary Materials.

**Table 1:** Summary of methodological characteristics, study design, and assessment instruments of the included studies.

Study (Country)	Design & Sample Size (Baseline/Final)	Age (Baseline → Follow-up)	Pragmatic Instrument (Informant)	Key Controls (IQ, Structural Language)	Methodological Quality/Notes
Ahmed (2023) (UK) (24)	Cross-sectional *N* = 200	11 years	Free-writing essay task (Direct assessment)	Yes (Verbal/Non-verbal IQ, Structural Language)	Moderate: Cross-sectional design limits causal inference.
Clegg (2005) (UK) (28)	Longitudinal *N* = 23/17	9 years → ∼36 years	Strange Stories; Bishop & Adams scheme (Direct)	Partial (Matched on Performance IQ; No structural lang control)	High: Very long follow-up (∼27 years) into adulthood.
Conti-Ramsden (2019) (UK) (31)	Longitudinal *N* = 242/168	7 years → 16 years	Children's Communication Checklist (CCC) (Teacher)	Yes (Grammar/Reception, WISC IQ)	High: Joint trajectory analysis of language and behavior.
Coplan (2009) (Canada) (34)	Longitudinal (Short-term) *N* = 167	6–7 years (8 months follow-up)	CASL Pragmatic Judgment test (Direct)	No (Controlled for parental education only)	Moderate: Short duration; correlational design.
Culpin (2018) (UK) (29)	Longitudinal (ALSPAC) *N* = 14,701/5,031	Birth → 16 years	SCDC (Parent)	No (Controlled for SES, Maternal MH)	High: Large population cohort; mediation analysis.
Done (2013) (UK) (22)	Longitudinal (NCDS) *N* = 15,145/166	11 years → Adult	Free-writing essay (Coherence) (Written)	Partial (Verbal IQ only; No structural language control)	High: Data linkage to psychiatric records.
Ketelaars (2010) (Netherlands) (25)	Cross-sectional *N* = 1,364	4 years	CCC (Teacher)	Yes (CCC Speech/Syntax subscales)	Moderate: Cross-sectional; reliance on single informant.
Law (2015) (UK) (30)	Longitudinal (ALSPAC) *N* = 13,992/2,926	Birth → 13 years	CCC Pragmatics Composite (Parent/Teacher)	Yes (Verbal & Non-verbal IQ)	High: Mediation analysis of social disadvantage.
Miranda (2023) (Spain) (20)	Longitudinal *N* = 52/45	7–11 years → ∼15 years	CCC-2 (Parent)	Yes (Structural subscales, IQ > 80)	Moderate: Separation of structural/pragmatic effects; no control for confounders in analysis.
Mok (2014) (UK) (32)	Longitudinal *N* = 242/139	7 years → 16 years	CCC (Teacher)	Partial (Grammar, WISC IQ tested; excluded from final model)	High: Discrete factor growth modelling.
Rodas (2017) (USA) (19)	Longitudinal (Short-term) *N* = 159	4–7 years (6 months follow-up)	CCC-2; SRS (Parent)	Yes (Syntax, IQ)	Moderate: Short-term SEM analysis.
Ryan (2015) (Australia) (21)	Longitudinal (TBI) *N* = 155	5–15 years → 24 months post-injury	TLC-E Making Inferences (Direct)	Yes (IQ, Digit Span)	Moderate: Clinical TBI sample; controlled for IQ and SES; attrition rate not reported.
Saul (2023) (UK) (26)	Cross-sectional *N* = 386	5–6 years	CCC-2 (Parent/Teacher)	Yes (CCC-2 Language Composite, WPPSI IQ)	Moderate: Cross-sectional; biased questionnaire return.
Skuse (2009) (UK) (27)	Short-term longitudinal (ALSPAC) *N* = 8,094	∼8 years	Social and Communication Disorders Checklist (SCDC) (Parent)	Yes (Full-scale IQ)	High: Large attrition; sex-by-IQ interaction analysis.
St Clair (2011) (UK) (33)	Longitudinal *N* = 242/103	7 years → 16 years	CCC (Teacher)	Partial (Gender only; IQ and SES examined but not included as covariates)	Low: Multilevel mixed-effects models; controlled for gender only; IQ and SES tested but not included as covariates; very high attrition (>50%).
Sullivan (2016) (UK) (23)	Longitudinal (ALSPAC) *N* = 7,659	9 years → 18 years	CCC (Parent)	Yes (Expressive Speech, Non-verbal IQ)	High: Multivariate probit modelling.

**Table 2:** Predictive impact of pragmatic abilities on psychopathological and adaptive trajectories: a synthesis of key findings.

Domain & Specific Outcome	Study (Author, Year)	Strength of Effect (Key Stats)	Specificity of Pragmatic Effect	Mediators or Moderators
1. EXTERNALIZING & BEHAVIORAL				
Hyperactivity & Conduct	Ketelaars (2010) (25)	RR up to 12.3 for total difficulties; strongly associated with hyperactivity.	High: Structural language (syntax) did not predict behavioral problems.	-
Behavioral Problems (General)	Law (2015) (30)	Unstandardized *β* ≈ −0.205. Significant effect on total difficulties at age 13.	Independent of verbal and non-verbal IQ.	Mediator: Pragmatic skills mediate the link between early social disadvantage and behavior.
Aggression & Rule-breaking	Ryan (2015) (21)	Correlations r = −0.33 (*p* = .003) at 24 months post-TBI.	Deficits pronounced even in IQ-matched groups.	Moderator: Age at insult and lesion location (frontal/temporal).
Behavioral & Academic	Saul (2023) (26)	β=−0.28 (*p* < .001). Unique variance in SDQ scores.	High: Prospectively predicts academic attainment and is associated with behavior independently of structural language and IQ.	-
2. INTERNALIZING & MOOD				
Anxiety vs. Externalizing	Rodas (2017) (19)	β = −0.601 (*p* < .001) for Anxiety vs. β = −0.067 (*p* < .001) for Externalizing.	High & Opposing: Higher structural skills predicted more anxiety; higher pragmatic skills predicted less anxiety.	-
Depressive Symptoms	Ahmed (2023) (24)	β = −0.22. More coherent essays are associated with fewer symptoms.	Association remained after controlling for structural language.	-
Suicidality & Self-Harm	Culpin (2018) (29)	RR = 2.14 for self-harm with suicidal intent.	Distinct from other autistic traits (e.g., repetitive behaviors).	Mediator: Depressive symptoms at age 12 partially mediate the risk.
Social Anxiety & Loneliness	Coplan (2009) (34)	sr2 up to.048. Pragmatics moderates the impact of shyness.	Specific “buffering” effect above other language skills.	Moderator: Pragmatic language protects shy children from developing social anxiety.
3. SOCIAL ADAPTATION & PEERS				
Peer Problems (Trajectories)	Mok (2014) (32)	OR = 2.53 of persistent peer problems if pragmatically impaired.	Significant effect established at age 7.	Protective: Prosocial behavior reduces the risk of negative trajectories.
Emotional & Peer Difficulties	Conti-Ramsden (2019) (31)	Mean diff = −8.60 (*p* = .004). Associated with severe joint trajectories.	High: No significant differences found for receptive/expressive language.	-
Social/Adaptive Skills	Miranda (2023) (20)	R2 up to.42. Predicts social and adaptive outcomes.	High: Only pragmatics (not structural) predicted behavioral problems.	-
Adult Social Adaptation	Clegg (2005) (28)	r = .52−.60. Correlates with employment and relationships at age 36.	Mixed: Structural language also correlated with adaptation.	Moderator: General language outcome severity (High vs. Low group).
4. MIXED/BROAD PSYCHOPATHOLOGY				
Functional Impairment (Hyperactivity, Conduct, Emotional)	Skuse (2009) (27)	β from.09 to.405 (Total Difficulties).	Independent of Full-scale IQ.	Moderator: Sex-by-Verbal IQ interaction (High VIQ protective for females only).
Behavioral, Emotional & Social	St Clair (2011) (33)	Significant associations (*p* < .01) across all three domains.	High: Pragmatics related to all domains; expressive language only to behavior.	-
5. SEVERE PSYCHOPATHOLOGY				
Psychotic Experiences	Sullivan (2016) (23)	OR up to 1.25 at ages 12 and 18.	High: Expressive speech/language ability was not associated with outcomes.	-
Schizophrenia	Done (2013) (22)	*p* < .02 (interaction), but main effect weak.	No evidence of specific pragmatic disorder in the pre-morbid phase.	Moderator: Gender (effect differs between males and females).

RR, risk ratio; OR, odds ratio; β, standardised regression coefficient; sr^2^, squared semi-partial correlation; R^2^, coefficient of determination; r, Pearson correlation coefficient; SDQ, Strengths and Difficulties Questionnaire; CCC, Children's Communication Checklist; SCDC, Social and Communication Disorders Checklist; CASL, Comprehensive Assessment of Spoken Language; TLC-E, Test of Language Competence-Expanded Edition; ASD, Autism Spectrum Disorder; DLD, Developmental Language Disorder; TBI, traumatic brain injury; ToM, theory of mind; IQ, intelligence quotient; SES, socioeconomic status; CI, confidence interval.

### 3.3 Synthesized findings

As described in section 2.7, findings are organized by five psychopathological domains and evaluated in terms of the strength of the predictive effect, the specificity of the pragmatic contribution, and the presence of identified mediators or moderators.

#### 3.3.1 Externalizing and behavioral problems

Four studies provided evidence on the predictive relationship between pragmatic language deficits and externalizing or behavioral outcomes. Ketelaars et al. (25) found that pragmatic competence, assessed with the CCC at age 4, was strongly associated with behavioral problems as measured by the SDQ, with crude, unadjusted risk ratios for total difficulties reaching 12.3 for girls and 11.3 for boys among children with pragmatic language impairment (no confidence intervals were reported). Notably, structural language abilities (speech and syntax) did not predict behavioral problems after accounting for pragmatic competence, supporting the specificity of the pragmatic effect.

Law et al. (30), drawing on the ALSPAC cohort, demonstrated that pragmatic language skills at age 9 partially mediated the relationship between early social disadvantage and adolescent behavioral problems at age 13 (unstandardized *β* ≈ −0.205, indirect effect ≈ −0.225). This mediating role was independent of verbal and nonverbal IQ and persisted after excluding children with an ASD diagnosis, indicating that pragmatic competence functions as a specific pathway linking socioeconomic adversity to behavioral maladjustment.

Ryan et al. (21) examined pragmatic communication recovery following pediatric traumatic brain injury. Poorer pragmatic skills (measured by the TLC-E Making Inferences subtest) at 24 months post-injury were associated with more frequent aggression, rule-breaking, and conduct problems (partial correlations ranging from r = −0.20 to r = −0.33, *p* = .003). The relationship between pragmatic deficits and externalizing behavior strengthened over time, and was moderated by age at insult and the location of brain pathology (frontal and temporal lesions).

Saul et al. (26), using the Surrey Communication and Language in Education Study (SCALES) cohort, reported that social-pragmatic skills at ages 5 to 6 were uniquely associated with behavioral difficulties (*β* = −0.28, *p* < .001), accounting for significant variance in SDQ scores even after controlling for structural language, socioeconomic status, and nonverbal cognitive ability.

#### 3.3.2 Internalizing and mood difficulties

Four studies addressed the predictive link between pragmatic deficits and internalizing outcomes, including anxiety, depression, suicidality, and self-harm. Rodas et al. (19) employed structural equation modeling in a sample of children with ASD aged 4 to 7 years and found that pragmatic language (modeled as a latent factor) was inversely related to child anxiety (*β* = −0.601, *p* < .001) and externalizing behaviors (*β* = −0.067, *p* < .001) over a six-month period. These coefficients should be compared with caution given that the anxiety outcome was a single observed measure whereas externalizing behaviors was a latent construct. An intriguing finding was that higher structural language skills predicted higher levels of anxiety (*β* = 0.019, *p* < .05), highlighting the distinct and opposing roles of pragmatic vs. structural language in the development of internalizing difficulties.

Ahmed et al. (24), analyzing free-writing essays from the NCDS cohort at age 11, found that children producing more coherent written narratives had fewer teacher-rated internalizing symptoms (*β* = −0.22). This association remained significant after controlling for structural language measures (mean length of utterance, lexical diversity, word count), verbal and nonverbal IQ, and socioeconomic status, suggesting a specific pragmatic effect related to the ability to organize and communicate relevant information.

Culpin et al. (29), using the ALSPAC cohort, examined the relationship between social communication traits in childhood and suicidal outcomes at age 16. Impaired social communication [measured by the Social and Communication Disorders Checklist (SCDC) at approximately age 7.6 years] was associated with a higher risk of self-harm with suicidal intent (RR = 2.14), suicidal thoughts, and suicidal plans. Depressive symptoms at age 12 partially mediated this association, suggesting a developmental pathway from early pragmatic difficulties through adolescent mood disturbance to suicidal behavior. No association was found for other autistic traits (e.g., repetitive behavior), supporting the specificity of the social communication component.

Coplan and Weeks (34) demonstrated that pragmatic language acted as a moderating protective factor. In a community sample of children aged 6 to 7 years, the association between shyness and socio-emotional difficulties (social anxiety, loneliness, asocial behavior) was present only for children with lower pragmatic language skills (sr^2^ up to.048). Children with higher pragmatic competence were buffered from the negative effects of shyness on adjustment.

#### 3.3.3 Social adaptation and peer relationships

Four studies examined the role of pragmatic deficits in predicting peer relationship problems and broader social adaptation. Mok et al. (32), using discrete factor growth modeling in the MLS cohort, identified four distinct trajectories of peer problems from age 7 to 16: low or absent problems, childhood-limited, childhood-onset persistent, and adolescent-onset. Pragmatic language difficulties at age 7 were associated with 2.53 times higher odds [95% CI (1.01, 6.35), *p* = .048] of following a persistent peer problems trajectory compared to a low or absent problems trajectory. Prosocial behavior emerged as a strong protective factor against negative peer trajectories.

Conti-Ramsden et al. (31), also drawing on the MLS cohort, used joint trajectory analysis (multivariate latent class growth model) and identified five distinct joint trajectories of emotional and peer difficulties from age 7 to 16. Pragmatic competence was significantly associated with trajectory group membership. The group with persistent, elevated levels of both emotional and peer difficulties had significantly lower pragmatic scores [mean difference = −8.60, 95% CI (−14.41, −2.80), *p* = .004] than other groups. Importantly, no significant differences were found for receptive or expressive language measures, indicating a specific pragmatic effect on the co-occurrence of emotional and social difficulties.

Miranda et al. (20) examined childhood language predictors of adolescent outcomes in a clinical sample of children with ASD. Pragmatic language, assessed with the CCC-2, significantly predicted social, adaptive, and behavioral outcomes in adolescence, explaining up to 42% of the variance (R^2^ up to.42). Structural language (specifically semantics) predicted prosocial behavior and socialization skills, but only pragmatic language predicted behavioral difficulties, supporting the differential predictive validity of the two language domains.

Clegg et al. (28) provided the longest follow-up data available in the included literature, tracking individuals with developmental language disorders from childhood (mean age 9 years) into mid-adulthood (approximately age 36). Pragmatic language scores in adulthood correlated significantly with social adaptation outcomes, including employment and interpersonal relationships (r = .52 to .60). Four of 17 participants developed serious psychiatric disorders, including two cases of schizophrenia. Although structural language also correlated with social adaptation, the prominence of pragmatic deficits in predicting functional outcomes was evident across multiple measures.

#### 3.3.4 Mixed or broad psychopathology

Two studies provided evidence on the relationship between pragmatic deficits and a broad range of psychopathological outcomes spanning multiple domains. Skuse et al. (27), using the ALSPAC cohort (*N* = 8,094), found that social communicative deficits (measured by the SCDC at approximately age 7 years 8 months) were significantly associated with functional impairment across all SDQ domains assessed by teachers approximately one year later. Standardized regression coefficients ranged from *β* = .090 for conduct problems and *β* = .093 for peer problems to *β* = .202 for hyperactivity and *β* = .405 for total difficulties (all *p* < .001). These associations were independent of full-scale IQ, sex, and maternal education. A significant sex-by-verbal IQ interaction was also identified: higher verbal IQ was a protective factor against social communication impairments in females but not in males with above-average verbal IQ.

St Clair et al. (33), using multilevel mixed-effects models in the MLS cohort (ages 7 to 16), found that pragmatic abilities were significantly associated with behavioral, emotional, and peer difficulties (b coefficients ranging from −0.03 to −0.06, all *p* < .01). Behavioral problems decreased from childhood to adolescence, emotional problems also decreased but remained elevated above normative levels, while peer problems increased over time. Pragmatic abilities were uniquely associated with all three domains, whereas expressive language and reading skills were only related to behavioral problems, and receptive language was not associated with any outcome domain. This pattern further supports the specificity of the pragmatic contribution to diverse psychopathological trajectories.

#### 3.3.5 Severe psychopathology: psychotic experiences and schizophrenia

Two studies examined the predictive relationship between pragmatic deficits and psychotic or severe psychiatric outcomes, yielding contrasting results. Sullivan et al. (23), using the ALSPAC cohort (*N* = 7,659), employed multivariate probit modeling and found that poorer pragmatic language at age 9, as measured by the CCC, was associated with psychotic experiences at ages 12 and 18 (OR up to 1.25) and with depression at age 18. This association was specific to pragmatic language: expressive speech and language ability was not associated with either outcome. The effect remained significant after controlling for gender, socioeconomic status, concurrent depression at age 9, nonverbal IQ, and autistic traits.

In contrast, Done and Leinonen (22), using data from the NCDS, found no evidence of pre-morbid pragmatic deficits in individuals who later developed schizophrenia. Adolescents who subsequently received a schizophrenia diagnosis did not show significant deficits in narrative coherence or cohesion at age 11 compared to matched controls, after controlling for IQ, gender, and socioeconomic status. The only noteworthy finding was a gender-moderated group effect [F(1,82) = 6.1, *p* < .02], but the main effect was weak (*p* = .03). These contrasting findings suggest that the relationship between pragmatic impairments and psychotic outcomes is less robust than the evidence for internalizing and externalizing disorders, and may depend on the type of psychotic outcome, the developmental timing of assessment, and methodological differences between studies.

### 3.4 Assessment of risk of bias

The methodological quality of the 16 included studies was assessed using the Newcastle-Ottawa Scale (NOS), adapted for longitudinal cohort studies. Overall, the evidence base demonstrated moderate to high methodological rigor: eight studies were rated as High Quality (NOS scores 7 to 9), seven as Moderate Quality (NOS scores 5 to 6), and one as Low Quality (NOS score of 4). The full NOS scoring for each study is reported in Supplementary Table S3. A complementary domain-level risk-of-bias appraisal of all 16 studies is provided in Supplementary Table S5. Temporal precedence was rated high risk in the three cross-sectional studies, and shared method variance was rated high where the same informant rated both the pragmatic predictor and the psychopathological outcome.

High-quality studies were typically large-scale longitudinal investigations based on well-established population cohorts such as ALSPAC, NCDS, and MLS, characterized by robust sampling procedures, validated measures for both pragmatic language and psychopathological outcomes, and appropriate control for confounding variables (e.g., IQ, gender, socioeconomic status). These features support the internal and external validity of their findings and reinforce the evidence for predictive relationships between early pragmatic deficits and later psychopathological outcomes.

Moderate-quality studies often relied on clinical or smaller community samples, presented limited control of confounders, or adopted cross-sectional or short-term longitudinal designs, which inherently reduce the strength of causal inference. Three studies with cross-sectional designs or short-term follow-ups (24–26) were retained in the final synthesis, as described in the Methods section, but their findings are interpreted with appropriate caution.

Several sources of potential bias were identified across the literature. First, high attrition rates were a recurring concern in the longitudinal studies, frequently exceeding 30% (30, 31, 33). Such attrition increases the risk of selection bias and may compromise the representativeness of follow-up data. Second, the reliance on informant-report checklists (CCC, SCDC, SDQ) for both predictor and outcome variables in many studies introduces the possibility of shared method variance, particularly when the same informant (parent or teacher) rated both pragmatic skills and behavioral outcomes. Third, not all studies controlled adequately for structural language ability or socioeconomic variables: Miranda et al. (20) included no confounders in the final analysis, and St Clair et al. (33) controlled only for gender. This incomplete adjustment potentially conflates pragmatic-specific effects with broader linguistic or environmental influences. Fourth, the geographical concentration of studies (11 of 16 from the UK, with heavy reliance on ALSPAC and MLS cohorts) raises questions about the independence of findings and limits the cross-cultural generalizability of the evidence base. Beyond their geographical concentration, the included studies are not statistically independent. Four studies draw on the ALSPAC cohort (23, 27, 29, 30), two on the NCDS (22, 24), and three on the Manchester Language Study (31–33). The three Manchester Language Study reports analyze essentially the same baseline sample of children, so they should not be read as independent replications, particularly for the emotional and peer-relationship outcomes. Conclusions are correspondingly more secure where they replicate across genuinely distinct cohorts, as for externalizing outcomes (the Dutch community sample, ALSPAC, and SCALES), than where they rest on a single cohort analyzed repeatedly.

Despite these limitations, the evidence base demonstrates satisfactory methodological rigor in many respects. The majority of studies employed validated instruments, controlled for key confounders, and adopted longitudinal designs of sufficient duration to examine predictive relationships. The consistency of findings across studies of varying methodological quality strengthens confidence in the overall pattern of results. Nonetheless, the identified sources of bias should temper conclusions about the strength and precision of the predictive relationships reported.

## 4 Discussion

### 4.1 Summary of main findings

The findings suggest that pragmatic competence may be a significant and specific predictor of a broad range of difficulties, particularly internalizing and externalizing disorders. Across multiple high quality studies, early pragmatic impairments were associated with increased risk for anxiety, depression, suicidality, attentional and conduct problems, and persistent peer relationship issues. These associations appeared robust even after controlling for structural language abilities and general cognitive functioning, a pattern that underscores the unique contribution of social communication skills to developmental trajectories and is discussed further in section 4.2. The evidence for a predictive link to psychotic disorders was less consistent: while one large cohort study found an association between poorer pragmatic language and psychotic experiences at ages 12 and 18 (23), another found no evidence of pre-morbid pragmatic deficits in individuals who later developed schizophrenia (22), suggesting that this relationship may be more complex or may emerge later in development.

Integrating the domain-level risk of bias judgments (Supplementary Table S5) with the sensitivity analyses (Supplementary Table S4), the strength of evidence varies across outcome domains. Evidence is strongest and most consistent for externalizing and behavioral problems and for internalizing and mood difficulties, where negative associations with pragmatic ability replicate across distinct cohorts and survive the exclusion of cross-sectional and clinical samples. Evidence for social adaptation and peer relationships is moderate but partly dependent on a single cohort, since three of the relevant studies derive from the Manchester Language Study. Evidence for broad or mixed psychopathology is more limited, resting on few studies and, under the strictest criterion, on a single large cohort. Evidence for severe psychopathology is inconsistent: one cohort study links poorer pragmatic language to later psychotic experiences, whereas a pre-morbid study finds no such relationship for schizophrenia. These gradations should be read alongside the conclusions stated below.

The broad predictive scope of pragmatic deficits is consistent with their tentative conceptualization as a transdiagnostic marker of vulnerability. Rather than linking to a single disorder, pragmatic impairments may be interpreted as tapping into a general liability that cuts across traditional diagnostic boundaries, an interpretation consistent with, though not directly tested against, the general psychopathology factor, or “p-factor.” The present findings suggest that the ability to use language effectively in social contexts may be a core component of this general vulnerability. Deficits in this domain may represent a final common pathway associated with various genetic and environmental risks, contributing to heterogeneous but interconnected psychopathological outcomes.

The transdiagnostic nature of pragmatics is further illuminated by the dimensional frameworks discussed in the Introduction. Within HiTOP, pragmatic deficits can be viewed as a lower order trait contributing to the development of higher order spectra, including internalizing, externalizing, and thought disorders (14). In the RDoC matrix, pragmatic communication maps onto fundamental constructs within the Social Processes domain and the Cognitive Systems domain (15). This framing shifts the focus from categorical diagnoses like Social (Pragmatic) Communication Disorder toward pragmatic ability as a dimensional, neurodevelopmental construct whose impairment has cascading effects on mental health. It should be emphasized that none of the included studies directly tested the general psychopathology factor, HiTOP spectra, or RDoC constructs. The transdiagnostic reading advanced here is therefore a theoretically generative hypothesis, consistent with the convergent pattern of findings, that future studies designed around these frameworks would need to test directly.

Several mechanisms may explain the pathway from pragmatic deficits to psychopathology. Consistent with the developmental cascades model (18), early pragmatic difficulties may initiate negative chains whereby impaired social communication hinders theory of mind development, provokes misinterpretations of social cues, and leads to peer rejection and social isolation (32). The present findings support this framework: depressive symptoms appear to mediate the link between communication difficulties and self-harm (29), while strong pragmatic skills can buffer shy children from the risk of developing social anxiety and loneliness (34), highlighting the role of communicative competence in building resilience.

### 4.2 Clinical and preventive implications

The findings of this review carry important clinical and preventive implications for child and adolescent psychiatry. The evidence that pragmatic deficits may precede the onset of psychosocial distress suggests that screening for these difficulties in early to middle childhood could be a potentially valuable but yet unvalidated tool for identifying at-risk individuals. The present evidence suggests that incorporating pragmatic language assessment into developmental surveillance may improve early identification of children vulnerable to multiple forms of psychopathology.

Interventions aimed at improving pragmatic competence may not only enhance communication but might also serve as a form of primary or secondary prevention, with the potential to influence developmental trajectories toward better social and emotional adjustment. The specificity of the pragmatic effect, documented across multiple studies in this review, is compatible with the hypothesis that targeted pragmatic interventions, rather than general language therapy alone, might be beneficial, although no intervention study was included in this review and this possibility remains untested. Given the transdiagnostic nature of pragmatic deficits, such interventions could benefit children across diverse clinical presentations, including those with developmental language disorders, autism spectrum disorder, attention deficit/hyperactivity disorder, and socially disadvantaged populations. Because the present review did not include intervention studies, these implications should be read as a research agenda rather than as support for the immediate adoption of pragmatic screening or intervention as a validated preventive strategy.

### 4.3 Limitations

The evidence base synthesized in this review has notable methodological strengths, including the predominance of large, well-characterized longitudinal cohorts and the consistent statistical control for key confounders such as IQ and structural language. However, several limitations must be considered, both at the level of the primary studies and at the level of the review itself.

Regarding the methodological quality of the primary studies, the Newcastle-Ottawa Scale assessment (see section 3.4 and Supplementary Table S3) identified several recurring sources of potential bias, including high attrition rates (in some cases exceeding 50%), shared method variance from reliance on informant-report checklists for both predictor and outcome variables, and incomplete control for structural language or socioeconomic variables in some studies.

At the level of the review, two additional limitations warrant consideration. First, there is significant heterogeneity in the measurement of pragmatic language across studies, with instruments ranging from parent-report checklists to direct assessment tools and researcher-designed coding schemes. While this diversity captures different facets of pragmatic competence, it complicates the comparison of findings and precludes quantitative synthesis through meta-analysis. A summary of the psychometric properties (internal consistency, test-retest reliability, and validity evidence) of the instruments used by the included studies, including their documented strengths and limitations, is provided in Supplementary Table S13 (35–49) to support the interpretation of the synthesised findings. The included studies also vary in the breadth of the construct captured by their pragmatic measures (Supplementary Table S8, continued). Several instruments, such as the Comprehensive Assessment of Spoken Language pragmatic judgment subtest, the Test of Language Competence inferencing subtest, and free-writing coherence coding, index pragmatic language relatively narrowly. Others, notably the Social and Communication Disorders Checklist used by Culpin et al. (29) and Skuse et al. (27), and the Social Responsiveness Scale used alongside the CCC-2 by Rodas et al. (19), tap broader social communication or autistic-trait dimensions. Because some outcomes concern peer relationships and social adjustment, this construct heterogeneity introduces a risk of overlap between predictor and outcome, and the findings for the social and peer domains in particular should be read with this in mind. Second, a major gap in the literature is the absence of studies explicitly testing for bidirectional effects; it is plausible that emerging psychopathology could also constrain or erode pragmatic skills over time, a hypothesis that remains unexplored in the current evidence base. The geographical concentration of studies in the United Kingdom, discussed in section 3.4, further limits cross-cultural generalizability.

Third, the present synthesis was restricted to peer-reviewed studies published in English, and grey literature, including dissertations, technical reports, conference proceedings, and unpublished cohort analyses, was not searched. This decision favours methodologically scrutinised evidence but introduces a risk of publication bias, since studies reporting statistically significant predictive associations between pragmatic deficits and psychopathological outcomes are more likely to reach publication than studies reporting null or weaker effects. The magnitude and direction of this bias cannot be quantified within the present design, and the predictive associations summarised here should therefore be considered as upper-bound estimates pending sensitivity analyses incorporating grey literature and registered-but-unpublished cohort outputs.

Fourth, two of the included studies (22, 24) operationalised pragmatic ability through free-writing essay tasks, in which children produced an extended written narrative that was subsequently coded for pragmatic features such as relevance, coherence, deictic reference, and conversational appropriateness. Although insightful, essay-based measures are inherently confounded by structural language competence, lexical productivity, and basic transcription and graphomotor skills, all of which constrain the surface form of the written output. Ahmed et al. (24) attempted to mitigate this confound by statistically controlling for mean length of utterance, lexical diversity, and total word count, whereas Done and Leinonen (22) controlled only for verbal IQ. Residual confounding from structural language and writing fluency therefore cannot be excluded, and findings derived from these tasks should be interpreted with appropriate caution.

Fifth, the inter-reviewer agreement reported for study selection (Cohen's kappa ≈ 0.8) refers to the overall inclusion decision rather than to the agreement on each individual eligibility criterion. The original screening records that would allow per-criterion kappa estimates were not retained in a retrievable form, and we therefore cannot quantify, for example, whether the two reviewers showed differential agreement on the age, design, pragmatic-focus, outcome, or methodological-quality criteria. Future updates of this review should preserve criterion-level rater records and report the corresponding agreement statistics, in line with PRISMA 2020 recommendations.

Sixth, the methodological quality scores derived from the Newcastle-Ottawa Scale and summarised in Supplementary Table S3 reflect the consensus reached after discussion between the two raters. Pre-consensus rater-level scores for the Selection, Comparability, and Outcome dimensions were not preserved in retrievable form. As a consequence, dimension-level inter-rater statistics cannot be computed retrospectively. The reported NOS scores should be interpreted as post-consensus values, and the absence of independent dimension-level ratings is acknowledged as a transparency limitation that future replications should address.

Seventh, the synthesis was tested for robustness under two stricter inclusion criteria than those specified in the protocol (Supplementary Table S4). Under Criterion A, which excludes studies whose primary baseline sample is characterised by a condition for which pragmatic impairment is a DSM-5-TR characteristic feature or by paediatric traumatic brain injury (three studies excluded), all five outcome-domain conclusions are preserved. Under Criterion B, which additionally excludes any clinical sample at baseline and retains only community-based cohorts and pre-morbid designs (seven studies excluded in total), four of the five outcome-domain conclusions remain robust; the conclusion on social adaptation and peer relationships is no longer assessable on the original taxonomy because all three remaining peer studies derive from the Manchester Language Study DLD cohort, and the conclusion on broad psychopathology rests on a single large study and is therefore flagged for cautious interpretation. Under Criterion C, which excludes the three studies with cross-sectional or concurrent-outcome designs (24–26) and retains only the 13 longitudinal studies, the externalizing and internalizing conclusions are preserved. The externalizing conclusion then rests on Law et al. (30) and Ryan et al. (21), and the internalizing conclusion on Rodas et al. (19), Culpin et al. (29), and Coplan and Weeks (34). The social adaptation, broad psychopathology, and severe psychopathology domains are unaffected, because none of their constituent studies is cross-sectional. Inferential breadth for the externalizing domain is reduced under this criterion, but the direction and significance of the associations are unchanged. The transparency of these analyses is intended to allow the reader to gauge the dependence of the synthesis on its inclusion criteria without requiring a re-execution of the search.

### 4.4 Future directions

Future research should aim to address the identified gaps. Longitudinal studies conducted in more diverse, cross-linguistic, and cross-cultural samples are needed to determine whether the predictive associations observed in predominantly Western, English-speaking populations generalize to other contexts. Future work should employ multimodal assessment batteries that combine questionnaires with objective behavioral tasks, discourse analysis, and neurocognitive measures. Furthermore, advanced statistical models, such as cross-lagged panel designs or random intercept cross-lagged panel models, should be used to explicitly test for bidirectional relationships between pragmatic development and mental health over time.

### 4.5 Conclusions

This systematic review provides converging evidence that pragmatic language competence appears to be a foundational skill for psychosocial development, and its impairment may represent an early and independent marker of vulnerability for a wide range of mental health problems. The longitudinal studies synthesized here demonstrate that the predictive validity of pragmatic deficits for internalizing, externalizing, and peer-related difficulties may be moderate to high, fulfilling, at the dimensional level, a key criterion for construct validity within a Kendlerian framework of epistemic iteration. The transdiagnostic nature of these associations calls for a shift away from categorical silo thinking and toward the integration of pragmatic language into dimensional models of psychopathology and routine developmental surveillance.

Moving forward, it is important that pragmatic language assessment is incorporated into both clinical research and public health initiatives. By recognizing early pragmatic difficulties not merely as a linguistic issue but as a potential predictor of future distress, clinicians and researchers can develop more effective and timely prevention programs. The future of this research lies in exploring these predictive relationships in more diverse global populations, leveraging multimodal and technologically advanced assessment methods, including AI-based language analysis, to capture the complexity of social communication, and ultimately, using this knowledge to foster resilience and positively influence developmental trajectories.

## Data Availability

The original contributions presented in the study are included in the article/Supplementary Material, further inquiries can be directed to the corresponding author.
