# Supplementary material for: Pragmatic language disorders as predictors of psychopathological trajectories in childhood and adolescence: a systematic review
Source: Front Child Adolesc Psychiatry. 2026 Jul 22;5:1860959. doi: 10.3389/frcha.2026.1860959 (PMC13438378; doi:10.3389/frcha.2026.1860959)
Supplement: Supplementary file 1 [file Datasheet1.docx]

**Supplementary Table S1 (S8). Comparative pragmatic profiles in DLD, ASD, ADHD, and pediatric TBI**

| **Clinical condition** | **Pragmatic profile** | **Underlying mechanism** | **Distinctive features** | **Key references** |
| --- | --- | --- | --- | --- |
| Developmental Language Disorder (DLD) | Pragmatic difficulties commonly secondary to structural language limitations: reduced topic maintenance, narrative incoherence, difficulty interpreting non-literal language, and poor inferencing in conversation | Primary impairment in structural language (syntax, semantics, phonology) with cascading effects on discourse organisation and conversational management | Pragmatic deficits track with grammar and lexical impairment; nonverbal social motivation is generally preserved; pragmatic profile is not the defining feature of the disorder | (1–3) |
| Autism Spectrum Disorder (ASD) | Severe and pervasive pragmatic impairment: deficits in inferential comprehension, social use of language, conversational reciprocity, and integration of contextual cues | Atypical social cognition, including theory of mind, joint attention, and social motivation; differences in the integration of linguistic and social-affective information | Pragmatic deficits accompanied by restricted, repetitive behaviours and atypical sensory processing; pragmatic deficits more severe and qualitatively more pronounced than in other clinical groups; sociocommunicative impairment is part of the diagnostic criteria | (3–5) |
| Attention-Deficit/Hyperactivity Disorder (ADHD) | Pragmatic difficulties especially in conversational initiation, topic management, turn-taking, and inhibition of off-topic contributions; verbose, tangential, or interruptive discourse style | Disturbances of attention regulation, executive control, response inhibition, and working memory rather than primary social-cognitive impairment | Profile is more variable than in ASD; structural language is largely preserved; pragmatic difficulties may improve with treatment of core ADHD symptoms; overlap with SPCD is documented but not equivalent | (3,6,7) |
| Pediatric traumatic brain injury (TBI) | Acquired pragmatic difficulties post-injury: reduced inferencing, narrative organisation, discourse coherence, and conversational appropriateness, with partial recovery over time | Acquired damage to frontal-temporal networks supporting executive functions and social cognition; injury-related disruption of integrative processes rather than developmental atypicality | Onset is linked to injury rather than developmental history; profile contingent on lesion location, age at insult, and injury severity; structural language is often relatively preserved | (8) |

**Note. Profiles are summarised at the population level; within-condition heterogeneity is substantial.**

**Supplementary Table S2 (S11). Search strategy by database**

| **Database** | **Search string / syntax** | **Filters and limits** | **Search date** | **Records retrieved** |
| --- | --- | --- | --- | --- |
| Google Scholar | Full primary query (three components combined with AND): ("pragmatic language impairment" OR "semantic-pragmatic disorder" OR "pragmatic language difficulties" OR "social (pragmatic) communication disorder" OR "pragmatic deficits" OR "pragmatic language skills") AND ("longitudinal" OR "follow-up" OR "prospective" OR "trajectory" OR "predict" OR "developmental outcome" OR "later adjustment" OR "future" OR "change over time") AND (child OR adolescent OR "early childhood" OR preschool OR "school-age") | Sorted by relevance; 20 results per page; the first 50 result pages (the platform ceiling) screened in descending order of relevance; relevance saturation reached on lower-ranked pages. | November to December 2025 | 1,000 |
| ScienceDirect | First component of the query only, owing to platform limits on Boolean operators: ("pragmatic language impairment" OR "semantic-pragmatic disorder" OR "pragmatic language difficulties" OR "social (pragmatic) communication disorder" OR "pragmatic deficits" OR "pragmatic language skills") | Publication years 1980 to 2025; English language; research-article document type. | November to December 2025 | 2,001 |
| PubMed | Full primary query (three components combined with AND), as above. | Publication years 1980 to 2025. | November to December 2025 | 71 |
| PsycINFO (APA PsycNet) | Any Field: SPCD OR pragmatic language impairment OR semantic-pragmatic disorder OR pragmatic language difficulties OR social (pragmatic) communication disorder, combined with Any Field: pragmatic deficits AND pragmatic language skills. | Peer-reviewed journals; Age groups Childhood (birth to 12 years) and Adolescence (13 to 17 years); Methodology Longitudinal Study. | November to December 2025 | 40 (5 duplicates; 35 excluded after title and abstract screening) |

*Note. The four databases together retrieved 3,112 records before deduplication. The first component of the query was deliberately broad to maximize sensitivity across the heterogeneous terminology historically used in this field, rather than to optimize precision. Procedures for duplicate removal and for flagging clearly ineligible records before manual screening are described in the Results (study selection) and summarized in the PRISMA flow diagram (Figure 1).*

**Supplementary Table S3 (S7).** Methodological Quality Assessment (Newcastle–Ottawa Scale)

| **Author (Year)** | **Study Design** | **Selection (0–4)** | **Comparability (0–2)** | **Outcome/Follow-up (0–3)** | **Total (0–9)** | **Quality Level** | **Key Notes** |
| --- | --- | --- | --- | --- | --- | --- | --- |
| Ahmed et al. (2023) | Cross-sectional (NCDS) | 3 | 2 | 1 | 6 | Moderate | Representative cohort (NCDS); validated measures for pragmatics (essay analysis) and psychopathology (Rutter, BSAG); multiple confounders controlled (IQ, SES); cross-sectional design limits causal inference. Use of extreme group and non standard pragmatic language measure push the evaluation on moderate. |
| Clegg et al. (2005) | Longitudinal (Clinical) | 3 | 2 | 3 | 8 | High | Longest follow-up (>25 years); small clinical sample (N=17) of men with severe DLD; multiple control groups (siblings, IQ-matched, NCDS); validated measures; all-male sample limiting generalizability. |
| Conti-Ramsden et al. (2019) | Longitudinal (MLS) | 4 | 2 | 2 | 8 | High | Representative DLD cohort (MLS); validated measures (CCC, Rutter, SDQ); 9-year follow-up; controlled for PIQ, gender, and parental mental health; attrition >30%. |
| Coplan & Weeks (2009) | Short-term longitudinal | 3 | 1 | 2 | 6 | Moderate | Community sample; validated measures (CASL, SASC-R); short follow-up (8 months); did not control for IQ/structural language. |
| Culpin et al. (2018) | Longitudinal (ALSPAC) | 4 | 1 | 2 | 7 | High | Large representative cohort (ALSPAC); validated measures; long follow-up; controlled for many confounders but not IQ/language; high attrition. |
| Done (2013) | Longitudinal (NCDS) | 4 | 2 | 3 | 9 | High | Representative cohort (NCDS); longitudinal design with long follow-up; validated outcome measure (PSE); controlled for IQ, gender, and SES; attrition <30%. |
| Ketelaars et al. (2010) | Cross-sectional | 3 | 2 | 1 | 6 | Moderate | Large community sample; validated measures (CCC, SDQ); controlled for structural language, gender, and SES; cross-sectional design limits predictive validity. |
| Law et al. (2015) | Longitudinal (ALSPAC) | 4 | 2 | 2 | 8 | High | Large representative cohort (ALSPAC); validated measures (CCC, SDQ); controlled for IQ, gender, and age; very high attrition. |
| Miranda et al. (2023) | Longitudinal (Clinical) | 2 | 0 | 3 | 5 | Moderate | Clinical ASD sample without comparison group; validated measures (CCC-2, SDQ, VABS); long follow-up with low attrition; no control for confounders in analysis. |
| Mok (2014) | Longitudinal (MLS) | 3 | 1 | 3 | 7 | High | Longitudinal design (MLS cohort); validated measures (CCC, SDQ, Rutter); long follow-up (9 years) with attrition <30%; did not control for IQ/structural language in the final model. |
| Rodas et al. (2017) | Short-term longitudinal | 2 | 1 | 3 | 6 | Moderate | Clinical ASD sample without comparison group; validated measures (CCC-2, SRS, CBCL); short follow-up (6 months); controlled for IQ but not other confounders. |
| Ryan et al. (2015) | Longitudinal (Clinical) | 2 | 2 | 2 | 6 | Moderate | Clinical TBI sample with a matched TD comparison group; validated measures (TLC-E, CBCL); 24-month follow-up; controlled for IQ, SES, and age; attrition rate not reported. |
| Saul et al. (2023) | Cross-sectional (SCALES) | 3 | 2 | 1 | 6 | Moderate | Representative cohort (SCALES); validated measures (CCC-2, SDQ); controlled for language, IQ, and SES; cross-sectional design limits predictive validity. |
| Skuse et al. (2009) | Short-term longitudinal (ALSPAC) | 3 | 2 | 2 | 7 | High | Representative cohort (ALSPAC); validated measures (SCDC, SDQ); 8-month follow-up; controlled for IQ, sex, and SES; short-term longitudinal design; significant data attrition for key analyses. |
| St Clair et al. (2011) | Longitudinal (MLS) | 2 | 0 | 2 | 4 | Low | Clinical SLI cohort (MLS) without comparison group; long follow-up (9 years) with validated measures (CCC, SDQ); controlled for gender only; IQ and SES tested but not included as covariates; very high attrition (>50%). |
| Sullivan et al. (2016) | Longitudinal (ALSPAC) | 4 | 2 | 2 | 8 | High | Representative cohort (ALSPAC); validated measures (CCC, PLIKSi, MFQ); long follow-up; controlled for IQ and multiple other confounders; high attrition rate. |

*Note. NOS = Newcastle-Ottawa Scale; NCDS = National Child Development Study; ALSPAC = Avon Longitudinal Study of Parents and Children; MLS = Manchester Language Study; SCALES = Surrey Communication and Language in Education Study; CCC = Children's Communication Checklist; CCC-2 = Children's Communication Checklist, second edition; SCDC = Social and Communication Disorders Checklist; SDQ = Strengths and Difficulties Questionnaire; CBCL = Child Behavior Checklist; PSE = Present State Examination; PLIKSi = Psychosis-Like Symptom Interview; MFQ = Moods and Feelings Questionnaire; VABS = Vineland Adaptive Behavior Scales; SRS = Social Responsiveness Scale; TLC-E = Test of Language Competence-Expanded Edition; ASD = Autism Spectrum Disorder; DLD = Developmental Language Disorder; SLI = Specific Language Impairment; TBI = traumatic brain injury; IQ = intelligence quotient; PIQ = performance IQ; SES = socioeconomic status; TD = typically developing.*

**Supplementary Table S4 (S10). Sensitivity analyses under two stricter inclusion criteria**

To assess the robustness of the synthesis under stricter inclusion criteria than those specified in the protocol, we report two sensitivity analyses. Criterion A excludes studies whose primary baseline sample is characterised by a clinical condition for which pragmatic impairment is a DSM-5-TR characteristic feature (autism spectrum disorder; Miranda et al., 2023; Rodas et al., 2017) or by paediatric traumatic brain injury (Ryan et al., 2015), reducing the synthesis to 13 studies. Criterion B is more conservative still and excludes any study with a clinical sample at baseline, retaining only studies based on community or general-population cohorts and pre-morbid designs targeting later schizophrenia outcomes; under criterion B the synthesis comprises 9 studies. The table below lists, for each outcome domain, the studies that remain available under each criterion together with the corresponding robustness of the conclusion.

| **Outcome domain** | **Studies remaining under Criterion A (n = 13)** | **Studies remaining under Criterion B (n = 9)** | **Robustness of the conclusion** |
| --- | --- | --- | --- |
| Externalising and behavioural problems | Ketelaars et al. (2010); Law et al. (2015); Saul et al. (2023). n = 3. | Ketelaars et al. (2010); Law et al. (2015); Saul et al. (2023). n = 3. | Robust under both criteria. The pattern of negative associations between pragmatic ability and externalising outcomes is replicated across distinct cohorts (CCC-Dutch, ALSPAC, SCALES) regardless of the criterion applied. |
| Internalising and mood difficulties | Ahmed et al. (2023); Coplan & Weeks (2009); Culpin et al. (2018). n = 3 (Rodas et al., 2017, excluded). | Ahmed et al. (2023); Coplan & Weeks (2009); Culpin et al. (2018). n = 3. | Robust under both criteria. The negative associations between pragmatic ability and internalising outcomes are preserved across NCDS, ALSPAC, and a community sample. Sullivan et al. (2016) is treated under the Severe psychopathology row (its primary outcome in the synthesis) and is not double-counted here. |
| Social adaptation and peer relationships | Mok et al. (2014); Conti-Ramsden et al. (2019); Clegg et al. (2005). n = 3 (Miranda et al., 2023, excluded). | None remains when peer outcomes are scored strictly under the original taxonomy of section 3.3, since the three remaining peer studies are all based on the Manchester Language Study DLD cohort. Two community-sample studies (Coplan & Weeks, 2009; Skuse et al., 2009) report effects on peer-relevant outcomes (loneliness, social anxiety, peer problems on the SDQ) and could be reassigned to this domain, in which case n = 2. | Robust under criterion A. Under criterion B the conclusion is no longer assessable on the original peer-specific taxonomy and rests on the cross-domain reassignment of two community-sample studies; the conclusion should therefore be flagged as uncertain under the strict criterion. |
| Mixed or broad psychopathology | Skuse et al. (2009); St Clair et al. (2011). n = 2. | Skuse et al. (2009). n = 1. | Robust under criterion A. Under criterion B the conclusion rests on a single large study (ALSPAC, N = 8,094); the direction and magnitude of the SCDC associations are unchanged but inferential breadth is reduced. |
| Severe psychopathology: psychotic experiences and schizophrenia | Sullivan et al. (2016); Done & Leinonen (2013). n = 2. | Sullivan et al. (2016); Done & Leinonen (2013). n = 2. | Robust under both criteria. Both studies use pre-morbid designs by construction and are unaffected by either criterion. |

Note. "Robust" indicates that the qualitative direction and statistical significance of the predictive associations are preserved under the corresponding criterion. "Reduced inferential breadth" indicates that the conclusion still holds in direction and significance but is supported by fewer independent studies. "No longer assessable" indicates that the corresponding outcome domain cannot be evaluated under the strict criterion using the original taxonomy of section 3.3.

**Criterion C (sensitivity analysis excluding the three cross-sectional studies)**

Criterion C, added in response to the reviewer comments, excludes the three studies with cross-sectional or concurrent-outcome designs (Ahmed et al., 2023; Ketelaars et al., 2010; Saul et al., 2023) and retains only the 13 longitudinal studies. For each outcome domain, the studies that remain available under Criterion C and the robustness of the conclusion are reported below.

| **Outcome domain** | **Studies remaining under Criterion C (n = 13)** | **Robustness of the conclusion** |
| --- | --- | --- |
| Externalising and behavioural problems | Law et al. (2015); Ryan et al. (2015). n = 2. | Robust in direction and significance. Inferential breadth is reduced relative to the full set, because the two cross-sectional contributors (Ketelaars et al., 2010; Saul et al., 2023) are removed. |
| Internalising and mood difficulties | Rodas et al. (2017); Culpin et al. (2018); Coplan & Weeks (2009). n = 3. | Robust. Only the cross-sectional study (Ahmed et al., 2023) is removed; the longitudinal evidence is unchanged. |
| Social adaptation and peer relationships | Mok et al. (2014); Conti-Ramsden et al. (2019); Miranda et al. (2023); Clegg et al. (2005). n = 4. | Robust. The domain is unaffected, because none of its constituent studies is cross-sectional. |
| Mixed or broad psychopathology | Skuse et al. (2009); St Clair et al. (2011). n = 2. | Robust. The domain is unaffected, because neither constituent study is cross-sectional. |
| Severe psychopathology: psychotic experiences and schizophrenia | Sullivan et al. (2016); Done & Leinonen (2013). n = 2. | Robust. Both studies use pre-morbid or prospective designs and are unaffected. |

**Supplementary Table S5 (S12). Domain-level risk of bias for the 16 included studies**

| **Study (year)** | **D1 Selection and participation** | **D2 Attrition** | **D3 Exposure measurement** | **D4 Outcome measurement** | **D5 Temporal precedence** | **D6 Confounding** | **D7 Shared method variance** |
| --- | --- | --- | --- | --- | --- | --- | --- |
| Ahmed (2023) | Moderate | Low | Moderate | Low | High | Low | Low |
| Clegg (2005) | Moderate | Moderate | Moderate | Low | Moderate | Moderate | Low |
| Conti-Ramsden (2019) | Low | Moderate | Low | Low | Moderate | Moderate | High |
| Coplan & Weeks (2009) | Moderate | Low | Moderate | Low | Moderate | High | Moderate |
| Culpin (2018) | Low | High | Moderate | Low | Low | Moderate | Low |
| Done & Leinonen (2013) | Low | Moderate | Moderate | Low | Low | Moderate | Low |
| Ketelaars (2010) | Moderate | Low | Moderate | Low | High | Moderate | High |
| Law (2015) | Low | High | Low | Low | Low | Low | High |
| Miranda (2023) | Moderate | Low | Low | Low | Low | High | Moderate |
| Mok (2014) | Moderate | Moderate | Low | Low | Moderate | Moderate | High |
| Rodas (2017) | Moderate | Low | Moderate | Low | Low | Moderate | High |
| Ryan (2015) | Moderate | Moderate | Low | Low | Low | Moderate | Low |
| Saul (2023) | Low | Low | Low | Low | High | Low | Moderate |
| Skuse (2009) | Low | High | Moderate | Low | Low | Low | Low |
| St Clair (2011) | Moderate | High | Low | Low | Low | High | High |
| Sullivan (2016) | Low | High | Low | Low | Low | Low | Low |

*Note. Each of the 16 included studies was rated Low, Moderate, or High risk of bias on seven domains. The scheme was adapted from the QUIPS tool for prognostic-factor reviews (Hayden et al., 2013) and extended, in response to the reviewer comments, with two domains of particular relevance to this literature: the temporal precedence of the pragmatic exposure relative to the psychopathological outcome (D5), and shared method variance between predictor and outcome (D7). Domain definitions: D1, sample selection and participation; D2, attrition and completeness of follow-up; D3, measurement of the pragmatic exposure, including instrument and control for structural language; D4, measurement of the psychopathological outcome, including instrument and informant; D5, temporal precedence of exposure relative to outcome; D6, control of confounding; D7, shared method variance between predictor and outcome and adequacy of the statistical analysis. Temporal precedence (D5) is rated High in the three cross-sectional studies (Ahmed et al., 2023; Ketelaars et al., 2010; Saul et al., 2023). Shared method variance (D7) is rated High where the same informant rated both the pragmatic predictor and the psychopathological outcome (Conti-Ramsden et al., 2019; Ketelaars et al., 2010; Mok et al., 2014; Rodas et al., 2017; St Clair et al., 2011) and Low where the predictor and outcome were derived from distinct sources (Ahmed et al., 2023; Clegg et al., 2005; Culpin et al., 2018; Done & Leinonen, 2013; Skuse et al., 2009; Sullivan et al., 2016).*

**Supplementary Table S6 (S1).** Study Identification

| **Authors / Year** | **Country** | **Study design** | **Follow-up duration** | **Sample size (baseline / final)** | **Age at baseline** | **Sex (% female)** | **Data source / cohort name** |
| --- | --- | --- | --- | --- | --- | --- | --- |
| Ahmed K, Flouri E & Vigliocco G (2023) | UK | Cross-sectional | N/A | 200 / 200 | 11 years | 44% | National Child Development Study (NCDS) |
| Clegg J, Hollis C, Mawhood L & Rutter M (2005) | UK | Longitudinal | ~27 years | 23 / 17 | Mean 9 years | 0% | Bartak et al. (1975) DLD cohort |
| Conti-Ramsden G, Mok P, Durkin K, Pickles A, Toseeb U & Botting N (2019) | UK | Longitudinal | 9 years | 242 / 168 | 7 years | 24% | Manchester Language Study (MLS) |
| Coplan RJ & Weeks M (2009) | Canada | Longitudinal | 8 months | 167 / 167 | 6-7 years | 50% | N/A |
| Culpin I, Mars B, Pearson RM, et al. (2018) | UK | Longitudinal | Up to 16 years | 14,701 / 5,031 | Birth | N/A | Avon Longitudinal Study of Parents and Children (ALSPAC) |
| Done DJ & Leinonen E (2013) | UK | Longitudinal | Up to 16 years | 15,145 / 166 | 11 years | N/A | National Child Development Study (NCDS) |
| Ketelaars MP, Cuperus J, Jansonius K & Verhoeven L (2010) | Netherlands | Cross-sectional | N/A | 1364 / 1364 | 4 years | 50% | N/A |
| Law J, Rush R, Clegg J, et al. (2015) | UK | Longitudinal | Up to 13 years | 13,992 / 2,926 | Birth | 50.3% | Avon Longitudinal Study of Parents and Children (ALSPAC) |
| Mok PL, Pickles A, Durkin K & Conti-Ramsden G (2014) | UK | Longitudinal | 9 years | 242 / 139 | 7 years | 25% | Manchester Language Study (MLS) |
| Miranda A, Berenguer C, Baixauli I & Roselló B (2023) | Spain | Longitudinal | ~5 years | 52 / 45 | 7-11 years | 7.7% | N/A |
| Rodas NV, Eisenhower A & Blacher J (2017) | USA | Longitudinal | ~6 months | 159 / 159 | 4-7 years | 18% | Smooth Sailing study |
| Ryan NP, Catroppa C, Beare R, et al. (2015) | Australia | Longitudinal | 24 months | 155 / 155 | 5-15 years | 36% | N/A |
| Saul J, Griffiths S & Norbury CF (2023) | UK | Cross-sectional with short-term prospective analysis | 1 year (Academic outcomes) | 386 / 386 | 5-6 years | 52% | Surrey Communication and Language in Education Study (SCALES) |
| Skuse DH, Mandy W, Steer C, et al. (2009) | UK | Short-term longitudinal | ~1 year | 8,094 / 8,094 | 7 years 8 months | 48.5% | Avon Longitudinal Study of Parents and Children (ALSPAC) |
| St Clair MC, Pickles A, Durkin K, & Conti-Ramsden G (2011) | UK | Longitudinal | 9 years | 242 / 103 | 7 years | 23.5% | Manchester Language Study (MLS) |
| Sullivan SA, Hollen L, Wren Y, et al. (2016) | UK | Longitudinal cohort study | 9 years | 7659 / 7659 | 9 years | 52.2% | Avon Longitudinal Study of Parents and Children (ALSPAC) |

*Note. NCDS = National Child Development Study; ALSPAC = Avon Longitudinal Study of Parents and Children; MLS = Manchester Language Study; SCALES = Surrey Communication and Language in Education Study; DLD = Developmental Language Disorder; SLI = Specific Language Impairment; ASD = Autism Spectrum Disorder; TBI = traumatic brain injury; N/A = not applicable.*

**Supplementary Table S7 (S1bis). Clinical diagnostic category and comparison groups for the 16 included studies**

| **Author (Year)** | **Clinical category (Population)** | **Comparison group(s) and N** |
| --- | --- | --- |
| Ahmed (2023) | General population (NCDS); analytic subsample with full essay data | Within-cohort variation in pragmatic essay scores; analytic N = 200 of approximately 17,400 NCDS members; no separate clinical comparison group |
| Clegg (2005) | Severe receptive Developmental Language Disorder (DLD), male-only clinical cohort | DLD N = 17 (final adult follow-up). Three matched comparison groups: non-language-disordered siblings of the DLD cohort (controls for shared family background); IQ-matched (IQM) adult controls without DLD; National Child Development Study (NCDS) reference subsample matched on childhood IQ and social class |
| Conti-Ramsden (2019) | Developmental Language Disorder (DLD) cohort (Manchester Language Study, MLS) | Within-DLD joint trajectory analysis on N = 242 (analytic N = 168 at age 16). Five empirically derived joint trajectory subgroups served as the analytic comparators; no external typically developing (TD) comparison group |
| Coplan (2009) | General community sample (Canada); unselected early elementary school children | N = 167 children aged 6–7 years recruited from grade 1 classrooms. No separate comparison group; pragmatic ability examined as a continuous moderator of the shyness-adjustment association |
| Culpin (2018) | General population (Avon Longitudinal Study of Parents and Children, ALSPAC) | Within-cohort comparisons across SCDC severity bands; baseline N = 14,701 with 5,031 retained for primary analyses; no separate clinical comparison group |
| Done (2013) | General population (NCDS); psychiatric outcomes derived from later linked records | Pre-morbid analytic sample N = 166 with available essays, comprising 102 individuals who later received a psychiatric diagnosis (Pre-Schizophrenia n = 22; Pre-Affective Psychosis n = 24; Pre-Non-Psychotic Psychiatric Disorders n = 56) and 64 matched controls (drawn from an initial subsample of N = 70 selected from the NCDS reference population and matched on gender, verbal IQ, and socioeconomic status; 64 of the 70 produced essays for analysis) |
| Ketelaars (2010) | General Dutch community sample of 4-year-olds | N = 1,364 children. Within-sample dimensional analysis; no separate clinical comparison group |
| Law (2015) | General population (ALSPAC) | Within-cohort variation; analytic N = 2,926 of 13,992 live births in the original cohort. Mediation analysis conducted within the cohort; no separate comparison group |
| Miranda (2023) | Autism Spectrum Disorder (ASD) clinical sample (Spain) | ASD N = 52 at baseline (45 retained at follow-up). No separate typically developing comparison group; longitudinal regression analyses conducted within the ASD sample |
| Mok (2014) | Developmental Language Disorder (DLD/SLI) cohort (MLS) | Within-DLD discrete-factor growth modelling on N = 242 (139 at follow-up). Four empirically derived peer-relations trajectory subgroups served as analytic comparators; no separate TD comparison group |
| Rodas (2017) | Autism Spectrum Disorder (ASD) clinical sample (Smooth Sailing Study, USA) | ASD N = 159 children aged 4–7 years. No separate comparison group; structural equation modelling within the ASD sample |
| Ryan (2015) | Pediatric mild-to-severe traumatic brain injury (TBI), clinical sample | TBI N = 112, stratified by age at insult: Middle Childhood (5–9 years; n = 41), Late Childhood (10–11 years; n = 39), Adolescence (12–15 years; n = 32). Group-matched typically developing (TD) comparison group N = 43, matched on age, gender, and socioeconomic status |
| Saul (2023) | General population (Surrey Communication and Language in Education Study, SCALES) | Population-based stratified random sample N = 386, aged 5–6 years. CCC-2 algorithms used to derive a putative SPCD subgroup (poor social-pragmatic skills with relatively preserved structural language and without autistic symptomatology) compared with a language-typical reference subgroup drawn from within the same SCALES sample |
| Skuse (2009) | General population (ALSPAC) | Within-cohort N = 8,094 with parent-rated SCDC. Severity-graded comparisons within the cohort; correlations with independently ascertained ASD diagnoses, cognitive ability, and teacher-rated maladaptive behaviour. No separate clinical comparison group |
| St Clair (2011) | Specific Language Impairment (SLI) cohort (MLS) | Within-SLI mixed-effects modelling on N = 242 at age 7, with 103 retained at age 16. No separate TD comparison group; trajectory analyses examine within-cohort change |
| Sullivan (2016) | General population (ALSPAC) | Within-cohort N = 7,659. Multivariate probit modelling examines pragmatic ability as a continuous predictor across the community sample; no separate clinical comparison group |

**Supplementary Table S8 (S2).** Summary of Pragmatic Measurement

| **Author** | **Main Pragmatic Domain** | **Instrument / Task** | **Language Modality** | **Age at Assessment** | **Control for Structural Language** | **Control for IQ/Executive Functions** |
| --- | --- | --- | --- | --- | --- | --- |
| Ahmed (2023) | Relevance, organization, deictic reference | Free-writing task (essay) with custom coding scheme | Written | 11 years | Yes (MLU, lexical diversity, word count) | Yes (verbal and non-verbal IQ) |
| Clegg (2005) | Inappropriacy, communicative intention, Theory of Mind | Bishop & Adams (1989) scheme on expressive language; Strange Stories; Eyes Task; Awkward Moments | Oral | ~24 years (Time 3; Pragmatics) & ~36 years (Time 4; ToM) | No | Yes (Performance IQ matched controls; partial correlations) |
| Conti-Ramsden (2019) | Inappropriate initiation, coherence, stereotyped conversation, use of context, rapport | Children’s Communication Checklist (CCC) | Oral (teacher-reported) | 11 years | Yes (Test for Reception of Grammar; CELF-R) | Yes (WISC-III UK) |
| Coplan (2009) | Pragmatic judgment | ‘Pragmatic judgment’ test of the CASL | Oral | 6–7 years | No | No (but parental education was controlled) |
| Culpin (2018) | Social communication | Social and Communication Disorders Checklist (SCDC) | Oral (parent-reported) | ~7.6 years (91 months) | No | No |
| Done (2013) | Narrative coherence, linguistic cohesion | Free-writing task (essay) with custom coding schemes | Written | 11 years | No | Yes (matched on verbal IQ) |
| Ketelaars (2010) | Inappropriate initiation, coherence, stereotyped conversation, use of context, rapport | Children’s Communication Checklist (CCC) | Oral (teacher-reported) | 4 years | Yes (CCC Speech and Syntax subscales) | No |
| Law (2015) | General pragmatic competence | Children’s Communication Checklist (CCC), pragmatics composite | Oral (Parent/Teacher Report) | 9 years | Yes (WISC-verbal scale) | Yes (WISC-nonverbal scale) |
| Miranda (2023) | Inappropriate initiation, stereotyped language, use of context, nonverbal communication | Children’s Communication Checklist Second Edition (CCC-2) | Oral (parent-reported) | 7-11 years | Yes (CCC-2 structural subscales) | Yes (IQ > 80) |
| Mok (2014) | Inappropriate initiation, coherence, stereotyped conversation, use of context, rapport | CCC and teacher report | Oral (teacher-reported) | 7 and 11 years | Yes (Test for Reception of Grammar; Bus Story Test; CELF-R) | Yes (Raven’s; WISC-III UK) |
| Rodas (2017) | Pragmatic language | CCC-2, Social Responsiveness Scale (SRS), Social Skills Improvement System (SSiS) | Oral (parent-reported) | 4-7 years | Yes (CASL syntax subscale, CCC-2 subscales) | Yes (WPPSI-III) |
| Ryan (2015) | Making inferences | Test of Language Competence-Expanded Edition (TLC-E), Making Inferences subtest | Oral | 5-15 years | No | Yes (WASI, Digit Span, TEA-Ch) |
| Saul (2023) | Social-pragmatic communication | Children’s Communication Checklist-2 (CCC-2) | Oral (parent and teacher reported) | 5–6 years | Yes (CCC-2 language composite) | Yes (WPPSI) |
| Skuse (2009) | Social reciprocity, verbal/nonverbal communication | Social and Communication Disorders Checklist (SCDC) | Oral (parent-reported) | ~7.7 years | Yes (minimal correlations with CCC subscales) | Yes (WISC-III) |
| St Clair (2011) | General Pragmatics | Children’s Communication Checklist (CCC) | Observed communicative behaviors (teacher report) | 11 years | Yes (expressive and receptive language measured at age 7) | Yes (Performance IQ measured at age 7) |
| Sullivan (2016) | Social communication | Children's Communication Checklist (CCC) | Oral (Parent-rated) | 9 years | Yes (Expressive Speech and Language score from CCC analyzed separately) | Yes (Non-verbal IQ) |

*Note. CCC = Children's Communication Checklist; CCC-2 = Children's Communication Checklist, second edition; SCDC = Social and Communication Disorders Checklist; CASL = Comprehensive Assessment of Spoken Language; TLC-E = Test of Language Competence-Expanded Edition; SRS = Social Responsiveness Scale; ToM = theory of mind; IQ = intelligence quotient; PIQ = performance IQ; MLU = mean length of utterance; WISC = Wechsler Intelligence Scale for Children; WPPSI = Wechsler Preschool and Primary Scale of Intelligence; ASD = Autism Spectrum Disorder; DLD = Developmental Language Disorder; SLI = Specific Language Impairment.*

**Supplementary Table S8 (continued) (S2 continued). Construct breadth of the pragmatic measures**

| **Study (year)** | **Pragmatic instrument** | **Construct breadth** |
| --- | --- | --- |
| Ahmed (2023) | Free-writing essay with custom pragmatic coding | Narrow linguistic pragmatics (discourse coherence and relevance) |
| Clegg (2005) | Bishop and Adams scheme; Strange Stories; Eyes task | Narrow linguistic pragmatics with theory-of-mind tasks |
| Conti-Ramsden (2019) | CCC pragmatic composite | Narrow linguistic pragmatics |
| Coplan & Weeks (2009) | CASL Pragmatic Judgment subtest | Narrow linguistic pragmatics |
| Culpin (2018) | SCDC | Broad social communication / autistic-trait screening |
| Done & Leinonen (2013) | Free-writing essay (coherence and cohesion) | Narrow linguistic pragmatics |
| Ketelaars (2010) | CCC pragmatic composite | Narrow linguistic pragmatics |
| Law (2015) | CCC pragmatics composite | Narrow linguistic pragmatics |
| Miranda (2023) | CCC-2 pragmatic subscales | Narrow linguistic pragmatics |
| Mok (2014) | CCC pragmatic composite | Narrow linguistic pragmatics |
| Rodas (2017) | CCC-2 with Social Responsiveness Scale (SRS) | Broad (autistic-trait screening, SRS) alongside narrow CCC-2 pragmatic |
| Ryan (2015) | TLC-E Making Inferences subtest | Narrow linguistic pragmatics (higher-order inferencing) |
| Saul (2023) | CCC-2 social-pragmatic | Narrow linguistic pragmatics |
| Skuse (2009) | SCDC | Broad social communication / autistic-trait screening |
| St Clair (2011) | CCC pragmatic composite | Narrow linguistic pragmatics |
| Sullivan (2016) | CCC pragmatic composite | Narrow linguistic pragmatics |

*Note. Construct breadth was classified as narrow linguistic pragmatics, for instruments that index pragmatic language relatively specifically (the CASL Pragmatic Judgment subtest, the TLC-E inferencing subtest, free-writing coherence coding, and the CCC and CCC-2 pragmatic composites), or as broad social communication or autistic-trait screening, for the Social and Communication Disorders Checklist (SCDC) and the Social Responsiveness Scale (SRS), which tap wider social-communication or autistic-trait dimensions. Because several outcomes concern peer relationships and social adjustment, this construct heterogeneity is relevant to interpreting the social and peer findings (see Limitations).*

**Supplementary Table S9 (già S3).** Psychopathology Outcomes

| **Author** | **Outcome type** | **Instrument used** | **Informant source** | **Age at outcome assessment** | **Main analysis type** | **Key results** | **Confounders controlled** | **Specificity of pragmatic effect** |
| --- | --- | --- | --- | --- | --- | --- | --- | --- |
| Ahmed (2023) | Internalising symptoms (depressive and anxiety) | Rutter’s Neurotic Scale; Bristol Social Adjustment Guide (BSAG) | Parent; Teacher | 11 years | Multiple linear regression | Children with more coherent essays had fewer teacher-rated internalising symptoms. Children providing more relevant information about future home-lives had fewer parent-rated internalising symptoms. | Cognitive ability (verbal/non-verbal IQ), socioeconomic status, sex, and structural language (MLU, vocabulary diversity, word count). | Associations for coherence and relevance of home-life information remained after controlling for structural language, suggesting a specific pragmatic effect. The effect of impure references was confounded by structural language. |
| Clegg (2005) | Social adaptation (employment, relationships); Schizotypal traits; Psychiatric morbidity | Schizotypal Personality Questionnaire (SPQ); Malaise Inventory; SEF interview; Records | Self; Informant; Records | ~36 years | Group comparisons (MANOVA/ANOVA); Correlations | DLD group showed severe deficits in social adaptation (employment, relationships) and higher schizotypal traits (social anxiety, no close friends). 4/17 developed serious psychiatric disorders (2 schizophrenia). Social adaptation correlated with pragmatic language scores (r=.52-.60). | Performance IQ, SES, Family background (siblings). | Pragmatic language scores correlated significantly with social adaptation, but so did structural language. Specificity not isolated via regression, but pragmatic deficits were prominent and associated with poor outcomes. |
| Conti-Ramsden (2019) | Emotional difficulties and peer problems | Rutter Children’s Behaviour Questionnaire; Strengths and Difficulties Questionnaire (SDQ) | Teacher | 7, 8, 11, 16 years | Joint trajectory analysis (multivariate latent class growth model) | Five distinct joint trajectories were identified. For just over half the sample, emotional and peer difficulties occurred together. The "persistent" problems group had high difficulties in both domains. Other groups showed adolescent-onset problems, resolving emotional problems, or increasing peer problems alone. | The analysis identified predictors for trajectory group membership, including: receptive language, expressive language, pragmatic language, prosociality, gender, and parental mental health. | Pragmatic competence was significantly associated with trajectory group membership. The group with persistent, high levels of both emotional and peer problems had significantly lower pragmatic scores than other groups. No significant differences were found for receptive or expressive language, indicating a specific pragmatic effect. |
| Coplan (2009) | Social anxiety, loneliness, asocial behavior | Social Anxiety Scale for Children—Revised (SASC-R); Loneliness and Social Dissatisfaction Questionnaire; Child Behaviour Scale (CBS) | Child; Teacher | 6-7 years | Hierarchical Regression Analysis | Pragmatic language acted as a protective factor. The association between shyness and socio-emotional difficulties (social anxiety, loneliness, asocial behavior) was present only for children with lower pragmatic language skills. | Parental education. | The study demonstrated a buffering effect of pragmatic language on the relationship between shyness and adjustment problems. The unique contribution of pragmatic language over and above other language skills was not assessed. |
| Culpin (2018) | Suicidal thoughts, plans, and self-harm | Self-report questionnaire based on Child and Adolescent Self-Harm in Europe Study (CASE) | Self-report | 16 years | Multinomial and modified Poisson regression; Structural equation modeling | Impaired social communication was associated with a higher risk of self-harm with suicidal intent, suicidal thoughts, and suicidal plans. No association was found for other autistic traits in the primary analysis. | Sex, ethnicity, socioeconomic status, maternal mental health, parental suicide attempt, childhood abuse/cruelty. | The study focused on social communication as a specific autistic trait. The findings suggest this trait is an important predictor of suicidality, distinct from other autistic traits like repetitive behavior or sociability. |
| Done (2013) | Schizophrenia, affective psychosis, non-psychotic psychiatric disorders | Present State Examination (PSE) CATEGO system | Psychiatrist (based on NHS records) | 16-27 years | ANOVA | Pre-morbid adolescents who later developed schizophrenia did not show significant deficits in narrative coherence or cohesion compared to matched controls. The hypothesis of limited or abnormal pragmatics was largely disproved. | IQ, gender, social and economic status. | The study found no evidence of a specific pragmatic language disorder in the pre-morbid phase of schizophrenia after controlling for IQ. |
| Ketelaars (2010) | Externalizing problems (hyperactivity, conduct), internalizing problems (emotional), peer problems, and lack of prosocial behavior. | Strengths and Difficulties Questionnaire (SDQ) | Teachers | 4 years (mean 4;11) | Hierarchical regression to predict behavioral problems from pragmatic competence, and MANOVA to compare behavioral problems between children with and without Pragmatic Language Impairment (PLI). | Pragmatic competence is a strong predictor of behavioral problems, while structural language abilities are not. Children with PLI show significantly more behavioral problems, especially hyperactivity and lack of prosocial behavior, with risk ratios for total difficulties up to 12.3 for girls and 11.3 for boys. | Gender (only). | Yes, the association was specific to pragmatic competence. Structural language abilities (speech and syntax) did not predict behavioral problems after accounting for pragmatic competence. |
| Law (2015) | Behavioral problems (emotional, conduct, hyperactivity, peer problems). | Strengths and Difficulties Questionnaire (SDQ). | Parent report. | 13 years. | Mediation analysis (linear regression). | Pragmatic language skills at 9 years partially mediate the relationship between early social disadvantage and adolescent behavioral problems at 13 years. This mediation effect was significant for total difficulties, emotional problems, conduct problems, hyperactivity, and peer problems. | Age, gender, verbal IQ, nonverbal IQ. | The mediating role of pragmatics was independent of verbal and nonverbal IQ. The findings remained consistent after excluding children with an ASD diagnosis. |
| Miranda (2023) | Prosocial behaviour, peer relationships, daily living skills, socialization skills, and behavioural difficulties. | School Social Behaviour Scales (SSBS); Strengths and Difficulties Questionnaire (SDQ); Vineland Adaptive Behaviour Scale (VABS-II). | Parent; Teacher. | 12-15 years. | Multiple linear regression. | Childhood pragmatic language significantly predicted social, adaptive, and behavioral outcomes in adolescence. Structural language (specifically semantics) predicted prosocial behavior and socialization skills. Stereotyped language and use of context were significant predictors for behavioral difficulties. | The analysis separated the effects of structural and pragmatic language to avoid multicollinearity. | Yes, only pragmatic language, not structural language, predicted behavioral problems. For social and adaptive skills, pragmatic language explained a larger portion of the variance than structural language. |
| Mok (2014) | Peer relations problems | Strengths and Difficulties Questionnaire (SDQ), Rutter Children’s Behaviour Questionnaire | Teacher | 7-16 years | Discrete factor growth modelling | Four trajectories of peer problems were identified (low-level, childhood-limited, childhood-onset persistent, adolescent-onset). Prosocial behavior was the strongest predictor of trajectory group membership. | The analysis investigated several potential predictors including language skills (expressive, receptive, pragmatic), emotional symptoms, conduct problems, hyperactivity, and prosocial behaviour. | Yes, pragmatic language difficulties at age 7 were associated with a 2.5 times higher odds of having persistent peer problems compared to children with low/no problems. |
| Rodas (2017) | Anxiety and Externalizing Behaviors | Child Behavior Checklist (CBCL) for anxiety and aggressive behaviors; Social Skills Improvement System (SSiS) for hyperactivity/inattention and bullying behaviors. | Parent | 4-7 years (The assessment was conducted at Time 2, approximately 6 months after the initial assessment). | Structural Equation Modeling (SEM) | Pragmatic language was inversely related to both child anxiety and externalizing behaviors over time. Higher structural language skills predicted higher levels of anxiety, but were not related to externalizing behaviors. | Child cognitive ability (IQ). | The study found that pragmatic language was a unique predictor of both anxiety and externalizing behaviors, even after accounting for structural language skills and cognitive ability. |
| Ryan (2015) | Externalizing behaviour (aggression, rule-breaking, conduct problems) | Child Behaviour Checklist (CBCL) | Parent | 6 and 24 months post-injury (ages 5-15 at baseline) | Pearson partial correlations | Poorer pragmatic communication at 24 months post-injury was associated with more frequent aggression, rule-breaking, and conduct problems. Reduced pragmatic communication at 6 months was associated with more frequent rule-breaking at 24 months. | Age at assessment, socio-economic status (SES). | The study links pragmatic deficits to behavioral issues but does not explicitly contrast this with structural language effects in the main correlation analyses. However, deficits were pronounced even in groups matched on IQ and executive functions, suggesting a primary pragmatic impairment. |
| Saul (2023) | Social, emotional, and behavioural problems; educational attainment | Strengths and Difficulties Questionnaire (SDQ); School achievement tests (SATS); Early Years Foundation Stage Profile | Parent; Teacher | 5-6 years | Prospective regression analysis | Children with social-pragmatic deficits showed poorer academic attainment and more behavioural problems. Social-pragmatic skills at Year 1 prospectively predicted academic development at Year 2 and uniquely predicted behaviour scores. | Socio-economic status, nonverbal cognitive ability, structural language skills. | Yes, social-pragmatic skills predicted behavioural and academic outcomes independently of structural language skills. |
| Skuse (2009) | Behavioral and emotional problems (hyperactivity, conduct problems, emotional symptoms, peer problems) | Strengths and Difficulties Questionnaire (SDQ) | Teacher | 8 years 4 months | Multivariable regression analysis | Social communicative deficits were significantly associated with functional impairment at school, including hyperactivity, conduct problems, peer problems, and emotional symptoms. | Sex, full-scale IQ, mother's highest educational level (socioeconomic status). | Yes, the association between social communication deficits (measured by SCDC) and behavioral outcomes remained significant after controlling for full-scale IQ. |
| St Clair (2011) | Behavioral, emotional, and social difficulties (hyperactivity, conduct, emotional, peer problems) | Strengths and Difficulties Questionnaire (SDQ) | Teacher | 7, 8, 11, 16 years | Longitudinal analysis (multilevel mixed-effects models) | Behavioral and emotional problems decreased from childhood to adolescence, while peer problems increased. Pragmatic abilities were related to all three domains of difficulty. | Gender. | Yes, pragmatic abilities were uniquely associated with behavioral, emotional, and social difficulties, whereas expressive language and reading skills were only related to behavioral problems, and receptive language was not related to any outcome. |
| Sullivan (2016) | Psychotic experiences and depression | Psychosis-Like Symptom interview (PLIKSi); Moods and Feelings Questionnaire (MFQ) | Interview; Parent (age 12); Self-report (age 18) | 12 and 18 years | Multivariate probit modelling | Poorer pragmatic language at age 9 was associated with psychotic experiences at ages 12 and 18, and with depression at age 18. | Gender, socio-economic status, concurrent depression at age 9, non-verbal IQ, autistic traits. | Yes, the association was specific to pragmatic language; expressive speech and language ability was not associated with either outcome. The effect was also stronger for psychotic experiences than for depression at age 12. |

*Note. SDQ = Strengths and Difficulties Questionnaire; CBCL = Child Behavior Checklist; BSAG = Bristol Social Adjustment Guide; MFQ = Moods and Feelings Questionnaire; PLIKSi = Psychosis-Like Symptom Interview; SPQ = Schizotypal Personality Questionnaire; SASC-R = Social Anxiety Scale for Children-Revised; VABS = Vineland Adaptive Behavior Scales; SEF = Social and Economic Functioning interview; PSE = Present State Examination; CCC = Children's Communication Checklist; SCDC = Social and Communication Disorders Checklist; MANOVA = multivariate analysis of variance; ANOVA = analysis of variance; SEM = structural equation modelling; OR = odds ratio; RR = risk ratio; β = standardised regression coefficient; r = Pearson correlation coefficient; CI = confidence interval; SES = socioeconomic status; IQ = intelligence quotient; ToM = theory of mind; ASD = Autism Spectrum Disorder; TBI = traumatic brain injury; DLD = Developmental Language Disorder.*

**Supplementary Table S10 (S4).** Mediators and Moderators

| **First Author (Year)** | **Tested mediators** | **Tested moderators** | **Evidence of mediation/moderation** |
| --- | --- | --- | --- |
| Ahmed (2023) | Not tested | Not tested | No |
| Clegg (2005) | Not tested | Language outcome severity (High vs Low Language Outcome groups) | Comparison of HLO/LLO groups showed that better language outcome was associated with better social adaptation (independent living, relationships), but not employment. |
| Conti-Ramsden (2019) | Not specified | Not specified | The study identifies pragmatic language ability, prosociality, and parental mental health as variables associated with joint trajectories of emotional and peer difficulties, but does not formally test for mediation or moderation. |
| Coplan (2009) | Not tested | Pragmatic language and gender | Yes, a significant moderating effect was found for pragmatic language. The association between shyness and adjustment difficulties was stronger for children with lower pragmatic language skills and was attenuated for those with higher skills. |
| Culpin (2018) | Depressive symptoms at 12 years | Not tested | Yes, depressive symptoms at 12 years partially mediated the association between social communication impairments in childhood and self-harm at 16 years. |
| Done (2013) | Not tested | Not tested | No |
| Ketelaars (2010) | Not tested | Not tested | No |
| Law (2015) | Pragmatic language skills | Not tested | Yes, pragmatic language skills partially mediate the relationship between social disadvantage and adolescent behavioral problems. |
| Miranda (2023) | Not tested | Not tested | No |
| Mok (2014) | Not tested | Not tested | No |
| Rodas (2017) | Not tested | Not tested | No evidence of mediation or moderation |
| Ryan (2015) | Not formally tested, but the study explores the relationship between pragmatic communication and externalizing behaviors within a diathesis-stress model. | Age-at-insult, injury severity, and location of brain pathology (frontal, temporal, corpus callosum lesions). | Yes. Persisting pragmatic language impairment is moderated by younger age at brain insult. Poorer pragmatic communication at 24 months is associated with frontal and temporal brain pathology. The relationship between pragmatic communication and externalizing behavior strengthens over time, suggesting a complex interaction rather than simple mediation. |
| Saul (2023) | Not tested | Not tested | No |
| Skuse (2009) | Not tested | Sex and verbal IQ | Yes, a significant sex-by-verbal IQ interaction was found. Higher verbal IQ is a protective factor against social communication impairments in females, but not in males with above-average verbal IQ. |
| St Clair (2011) | Not tested | Not tested | No |
| Sullivan (2016) | Not tested | Not tested | No evidence of mediation or moderation was tested. |

*Note. HLO = High Language Outcome; LLO = Low Language Outcome; ASD = Autism Spectrum Disorder; DLD = Developmental Language Disorder; PLI = Pragmatic Language Impairment; SLI = Specific Language Impairment; ToM = theory of mind; MH = mental health; TBI = traumatic brain injury.*

**Supplementary Table S11 (S5).** Methodological and Predictive Validity Assessment

| **Article** | **Level of longitudinal validity (Kendler’s framework)** | **Directionality tested (bidirectional models)** | **Overall methodological quality** | **Authors’ stated limitations** | **Theoretical implications** |
| --- | --- | --- | --- | --- | --- |
| Ahmed (2023) | Low (cross-sectional study) | No | Not specified | The findings are preliminary and the coding scheme needs refinement with contemporary child samples. The study is cross-sectional, which limits causal inference. | Coherent communication may act as a protective factor against emotional difficulties. Children's narratives about family life could be a useful indicator of their emotional state. |
| Clegg (2005) | High (longitudinal study with ~27-year follow-up) | No (unidirectional prediction) | High | Small sample size (N=17); High attrition; Male only sample; Pragmatics assessed in early adulthood, not childhood; Retrospective comparisons. | Persistent pragmatic deficits in DLD are associated with poor social adaptation and psychiatric morbidity in adulthood. Supports the link between DLD and broader psychosocial difficulties independent of IQ. |
| Conti-Ramsden (2019) | High (longitudinal study from age 7 to 16) | No (the study uses joint trajectory analysis to model parallel development, but discusses bidirectional causality as a possibility) | Not specified | Use of different measures at different ages, sample attrition, lack of standardized clinical measures for parental mental health, and a small proportion of female participants. | Development in children with DLD is heterogeneous. Pragmatic language competence is associated with the joint trajectory of emotional and peer problems. Prosociality and parental mental health are also important factors. |
| Coplan (2009) | Moderate (short-term longitudinal study over one school year) | No | Not specified | Correlational design limits causal inference; short duration; other language abilities not controlled for; potential for performance vs. competence issues in testing shy children. | Pragmatic language skills act as a buffer, attenuating the link between shyness and socio-emotional difficulties (social anxiety, loneliness, social withdrawal). |
| Culpin (2018) | High (longitudinal study to age 16) | No (mediation analysis was performed, but not bidirectional models) | Not specified | Underpowered for ASD diagnosis effects; potential for selection bias/attrition; self-report limitations for suicidal intent, especially in individuals with autistic traits. | Social communication impairments are a key autistic trait related to suicidality. Depression in early adolescence is a partial mediator, suggesting that addressing mental health needs is a potential preventive strategy. |
| Done (2013) | High (longitudinal birth cohort study from age 11 to adulthood) | Unidirectional | High | The data linkage approach may miss less severe cases of mental disorder. | Pragmatic language deficits are not a stable, early developmental marker for schizophrenia but likely emerge closer to the onset of the illness, either in the prodromal phase, post-onset, or as a state-dependent feature. |
| Ketelaars (2010) | Low (cross-sectional study) | No | Not specified | Use of teacher questionnaires for both language and behavior, which may introduce bias. The study's cross-sectional design limits causal inference. The CCC is used as the sole instrument for identifying PLI. | Pragmatic competence is a strong predictor of behavioral problems, more so than structural language skills. Early assessment of pragmatic competence could serve as a marker for detecting children at risk for behavioral issues and developmental disorders like autism or ADHD. |
| Law (2015) | High (longitudinal birth cohort study from birth to age 13) | No | High | Mediation analysis does not conclusively establish causality; potential for measurement error in the assessment tools; attrition is a recognized issue in the longitudinal cohort. | Pragmatic language skills are a key mediator between early social disadvantage and adolescent behavior. Interventions targeting pragmatic skills may be effective in reducing adolescent behavioral problems, especially for children from disadvantaged backgrounds. |
| Miranda (2023) | High (longitudinal study from childhood to adolescence) | No | Not specified | Reliance on parent/teacher questionnaires; small sample size; limited generalizability due to participant characteristics (mid-range IQ, mostly male); exclusion of family/school context factors and interventions. | Both structural and pragmatic language skills in childhood are significant predictors of social, adaptive, and behavioral outcomes in adolescents with ASD. Pragmatic language, in particular, is a strong predictor of later difficulties. |
| Mok (2014) | High (longitudinal study from age 7 to 16) | No | Not specified | No typically developing comparison group; reliance on teacher reports; sample from language units may not generalize to community samples with milder SLI. | Children with SLI show distinct trajectories of peer relations. Pragmatic language difficulties and emotional problems are risk factors for poor outcomes, while prosocial behavior is a strong protective factor. |
| Rodas (2017) | Moderate (short-term longitudinal study over one school year) | No (unidirectional predictive model) | Not specified | No clinical diagnosis of anxiety; reliance on parent-report; one pragmatic language measure was broad; sample may not be representative (higher income/education). | Pragmatic language deficits longitudinally predict both anxiety and externalizing behaviors in children with ASD. In contrast, higher structural language skills predict higher anxiety but not externalizing behaviors. |
| Ryan (2015) | High (longitudinal study over 24 months) | No | High | Inability to evaluate the contribution of family environment due to colinearity; possibility that deficits are partially attributable to executive function impairments; recovery in adolescents may not extend to other high-level cognitive functions. | Supports a critical period model, where TBI during middle childhood leads to persistent pragmatic language deficits. The strengthening relationship between pragmatic deficits and externalizing behavior over time supports a diathesis-stress model. |
| Saul (2023) | Moderate (Short-term predictive validity) | Unidirectional (Pragmatics → Academic/Behavior) | High | Biased questionnaire return (higher SES, lower language difficulties); no independent assessment of autism symptoms; CCC-2 is not a diagnostic instrument and its factor structure had limitations. | 'Pure' SPCD is extremely rare. Social-pragmatic deficits are commonly associated with structural language problems and autistic traits, suggesting these are intertwined competencies on a continuum. The exclusionary criteria of DSM-5 may be a "false dichotomy" leading to underidentification. |
| Skuse (2009) | High (longitudinal data from a large population cohort) | No (unidirectional predictive model) | High | SCDC does not measure all autistic traits (e.g., RRBs); potential for missed ASD cases in ascertainment; missing IQ data for a subset of children, particularly those with ASD. | Social communicative deficits are continuously distributed in the population. Even subclinical traits are associated with functional impairments (e.g., conduct problems). A sex-by-verbal IQ interaction exists, where high verbal IQ is protective for females but not for males with above-average verbal IQ. |
| St Clair (2011) | High (longitudinal study from age 7 to 16) | No (unidirectional predictive model) | High | The participant group is referred to as having a "history of SLI" and may not meet strict criteria throughout development; single instruments were used to assess each domain. | There are distinct developmental trajectories for different difficulties. Behavioral problems decrease to normative levels by adolescence, while emotional problems decrease but remain elevated. Social (peer) problems increase over time. Pragmatic abilities were related to behavioral, emotional, and social outcomes, whereas expressive language and reading were only related to behavioral problems. |
| Sullivan (2016) | High (longitudinal study from age 9 to 18) | No | High | No PE measure before age 9; communication ability might affect PE ratings; limited ESL measure; reliance on parental reports for language; lack of ethnic diversity in the cohort. | Poor pragmatic language in middle childhood precedes adolescent psychotic experiences and depression. Interventions aimed at improving pragmatic language skills may reduce the incidence of adolescent psychopathology. |

*Note. DLD = Developmental Language Disorder; SLI = Specific Language Impairment; ASD = Autism Spectrum Disorder; TBI = traumatic brain injury; SPCD = Social (Pragmatic) Communication Disorder; CCC = Children's Communication Checklist; CCC-2 = Children's Communication Checklist, second edition; SCDC = Social and Communication Disorders Checklist; SDQ = Strengths and Difficulties Questionnaire; PSE = Present State Examination; IQ = intelligence quotient; SES = socioeconomic status; ESL = English as a Second Language; RRBs = restricted, repetitive behaviours; PE = psychotic experiences; DSM-5 = Diagnostic and Statistical Manual of Mental Disorders, fifth edition.*

**Supplementary Table S12 (S6).** Predictive and Trajectory Analysis

| **Author (Year)** | **Pragmatic Stimulus Type** | **Analytical Unit** | **Statistical Model** | **Effect Size / CI** | **Domain Predicted** | **Direction of Association** | **Transdiagnostic Factor** | **Trajectory Pattern** | **Contextual Controls** | **Bias Risk** |
| --- | --- | --- | --- | --- | --- | --- | --- | --- | --- | --- |
| Ahmed (2023) | Free-writing task (essay) | Narrative (relevance, coherence, references) | Multiple linear regression | β = -0.22 | Internalising | Pragmatics → Psychopathology | IQ, structural language | Cross-sectional | SES, gender, parental education, overcrowding | Cross-sectional design |
| Clegg (2005) | Expressive language (Bishop & Adams); ToM tasks | Composite scores; Inappropriacy score | Group comparisons (MANOVA/ANOVA); Correlations | r = .52–.60 (Pragmatics-Social Adaptation) | Social adaptation, Psychiatric morbidity | Pragmatics → Psychopathology | Language outcome severity | Longitudinal (adult plateau) | IQ (matched), SES, family | Small sample, attrition |
| Conti-Ramsden (2019) | Teacher report (CCC) | Item composite | Latent class growth model | Mean diff = −8.60 (95% CI [−14.41, −2.80]), t(71)=−8.60, p=.004 | Emotional + Peer | Pragmatics → Psychopathology | Prosociality, parental MH | Five distinct joint trajectories | SES, gender, language | Attrition bias |
| Coplan (2009) | Pragmatic judgment test (CASL) | Composite score | Hierarchical Regression | sr² up to .048 | Social/adaptive | Pragmatics moderates Shyness → Psychopathology | Shyness | Short-term longitudinal | Parental education, gender | Correlational design |
| Culpin (2018) | Parent-report (SCDC) | Social communication trait (dichotomized) | Multinomial/Poisson regression | RR = 2.14 (suicidal self-harm) | Social/adaptive (self-harm) | Pragmatics → Psychopathology | Depressive symptoms | Longitudinal | SES, gender, maternal MH, abuse | Attrition bias |
| Done (2013) | Free-writing task (essay) | Narrative (coherence, cohesion) | ANOVA | F (1,82)=6.1, p <.02 (group×gender); main p =.03; no standardized β/OR | Psychotic | Pragmatics → Psychopathology (weak, gender-moderated) | IQ (controlled), gender | Longitudinal (risk factor) | SES, gender | Data linkage may miss cases |
| Ketelaars (2010) | Teacher-report (CCC) | Composite score | Hierarchical regression | RR up to 12.3 | Mixed (Ext, Int, Peer) | Pragmatics → Psychopathology | Not tested | Cross-sectional | SES, gender | Informant bias |
| Law (2015) | Parent/teacher report (CCC) | Composite score | Linear regression (mediation) | unstandardized β ≈ –.205, indirect = –.225 | Behavioral problems | Social disadvantage → Pragmatics → Behavior | Social disadvantage | Partial mediation | IQ, SES, gender | Attrition bias |
| Miranda (2023) | Parent-report (CCC-2) | Subscale scores | Multiple linear regression | R² up to .42 | Social/adaptive, Behavioral | Pragmatics → Psychopathology | Not tested | Longitudinal | Not reported | Small sample size |
| Mok (2014) | Teacher-report (CCC) | Composite score | Discrete factor growth model | OR = 2.53, 95% CI [1.01, 6.35], p = .048 (persistent peer problems associated with PLI) | Peer problems | Pragmatics → Psychopathology | Prosociality | Four latent trajectories identified: low/no, limited, persistent, and adolescent-onset peer problems. | Not reported | Sample not generalizable |
| Rodas (2017) | Parent-report (CCC-2, SRS) | Composite score | Structural Equation Modeling | β = .019 (p < .05, structural → anxiety); β = –.601 (p < .001, pragmatic → anxiety); β = –.067 (p < .001, pragmatic → externalizing) | Internalizing, Externalizing | Pragmatics → Psychopathology | IQ | Follow-up over 6 months (T1–T2) within a 2-wave longitudinal SEM. | Not reported | Informant bias |
| Ryan (2015) | Making Inferences task (TLC-E) | Standard score | Pearson partial correlations | r = –.20 (CC, p=.04) – –.33 (Rule-breaking, p=.003) | Externalizing | Pragmatics → Psychopathology | Injury severity | Longitudinal (diathesis-stress) | SES | Colinearity with family env. |
| Saul (2023) | Parent/teacher report (CCC-2) | Composite score | Regression analysis | β = –0.28 (p < .001) → unique variance in SDQ after controlling for language, SES, IQ | Behavioral, Social/adaptive | Pragmatics → Psychopathology | Structural language, IQ | Short-term prospective (Year 1 → Year 2) | SES | Biased questionnaire return |
| Skuse (2009) | Parent-report (SCDC) | Composite score | Multivariable regression | β = .090 (CP), .093 (PP), .202 (Hyp), .405 (Total Difficulties), all p < .001 | Mixed | Pragmatics → Psychopathology | Sex, full-scale IQ | Short-term longitudinal (ALSPAC) | SES | Missing IQ data |
| St Clair (2011) | Teacher-report (CCC) | Composite score | Multilevel mixed-effects models | b = –.04 (Hyp), –.05 (Cond), –.03 (Emot), –.06 (Peer & Total), all p < .01 | Mixed | Pragmatics → Psychopathology | Not tested | Longitudinal (distinct trajectories) | Gender | Sample selection bias |
| Sullivan (2016) | Parent-report (CCC) | Composite score | Multivariate probit modelling | OR up to 1.25 | Psychotic, Internalizing | Pragmatics → Psychopathology | Autistic traits, IQ | Longitudinal | SES, gender | Informant bias |

*Note. CCC = Children's Communication Checklist; CCC-2 = Children's Communication Checklist, second edition; SCDC = Social and Communication Disorders Checklist; CASL = Comprehensive Assessment of Spoken Language; TLC-E = Test of Language Competence-Expanded Edition; SRS = Social Responsiveness Scale; SDQ = Strengths and Difficulties Questionnaire; β = standardised regression coefficient; OR = odds ratio; RR = risk ratio; sr² = squared semi-partial correlation; R² = coefficient of determination; r = Pearson correlation coefficient; CI = confidence interval; SEM = structural equation modelling; T1 = Time 1; T2 = Time 2; CC = conduct problems; PP = peer problems; Hyp = hyperactivity; Cond = conduct; Emot = emotional; Ext = externalising; Int = internalising; IQ = intelligence quotient; SES = socioeconomic status; MH = mental health; PLI = Pragmatic Language Impairment; SLI = Specific Language Impairment; TBI = traumatic brain injury.*

**Supplementary Table S13 (S9). Psychometric properties of the instruments used in the included studies**

| **Instrument** | **Construct measured** | **Internal consistency (Cronbach's α)** | **Test-retest reliability** | **Validity evidence** | **Use in included studies** | **Primary references** |
| --- | --- | --- | --- | --- | --- | --- |
| Children's Communication Checklist (CCC) and CCC-2 | Pragmatic and structural language; communicative competence in everyday contexts | Generally good across subscales; the original CCC pragmatic composite reported α values around 0.80 in the validation sample | Adequate over short intervals in the validation samples reported in the original development paper | Construct validity supported by factor structure separating pragmatic from structural subscales; concurrent validity with clinical diagnoses of communication disorder | Conti-Ramsden 2019; Ketelaars 2010; Law 2015; Miranda 2023; Mok 2014; Rodas 2017; Saul 2023; St Clair 2011; Sullivan 2016 | (3, 35) |
| Social and Communication Disorders Checklist (SCDC) | Social-communicative competence as a screening dimension | High internal consistency reported in the original ALSPAC validation | Acceptable over short intervals in the original validation work | Discriminative validity for ASD clinical diagnoses; correlations with maladaptive behaviour and cognitive ability | Culpin 2018; Skuse 2009 | (36) |
| Comprehensive Assessment of Spoken Language (CASL), Pragmatic Judgment subtest | Pragmatic judgement and contextually appropriate language use | Reported as adequate in the test manual | Reported in the test manual | Construct and concurrent validity established via the test development programme | Coplan 2009 | (48) |
| Test of Language Competence-Expanded Edition (TLC-E), Making Inferences subtest | Higher-order language and inferencing | Reported in the test manual | Reported in the test manual | Construct validity established for higher-order language abilities; sensitivity to acquired language disturbance | Ryan 2015 | (49) |
| Free-writing essay tasks with custom pragmatic coding | Discourse coherence, relevance, organisation, deictic reference and conversational appropriateness in written narrative | Inter-rater agreement reported within the original studies; no standardised internal consistency available | Not standardised; assessed within studies through internal consistency of coding categories and inter-rater reliability | Construct validity supported by associations with independently measured pragmatic ability; criterion validity limited | Ahmed 2023; Done 2013 | (13,14) |
| Strengths and Difficulties Questionnaire (SDQ) | Behavioural and emotional difficulties (conduct, hyperactivity, emotional, peer, prosocial) | Good across subscales in published normative work | Acceptable in published normative work | Convergent validity with CBCL and Rutter scales; widely used in epidemiological samples | Conti-Ramsden 2019; Culpin 2018; Law 2015; Miranda 2023; Mok 2014; Saul 2023; Skuse 2009; St Clair 2011 | (37) |
| Child Behavior Checklist (CBCL) | Internalising and externalising behavioural problems | High in published normative work | Strong over short intervals in published normative work | Strong convergent validity across informant versions and concurrent validity with clinical diagnoses | Rodas 2017; Ryan 2015 | (38) |
| Rutter Children's Behaviour Questionnaire and Bristol Social Adjustment Guide (BSAG) | Behavioural and adjustment difficulties (older epidemiological instruments) | Acceptable in original epidemiological work | Reasonable in original epidemiological work | Concurrent validity with later instruments such as SDQ and CBCL | Ahmed 2023; Conti-Ramsden 2019; Mok 2014 | (43) |
| Mood and Feelings Questionnaire (MFQ) | Depressive symptoms | Good in adolescent samples in published validation work | Acceptable in adolescent samples in published validation work | Concurrent validity with diagnostic interviews for major depressive disorder | Sullivan 2016 | (39) |
| Psychosis-Like Symptom Interview (PLIKSi) | Psychotic experiences in adolescence | Inter-rater reliability and discriminative validity reported in the ALSPAC psychosis programme | Reasonable in ALSPAC validation work | Discriminative validity for clinical psychotic disorders | Sullivan 2016 | (40) |
| Schizotypal Personality Questionnaire (SPQ) | Schizotypal personality features | Good in published validation samples | Reasonable in published validation samples | Discriminative validity for schizotypal traits | Clegg 2005 | (41) |
| Social Anxiety Scale for Children-Revised (SASC-R) | Social anxiety in children | Good in published validation samples | Acceptable in published validation samples | Convergent validity with related anxiety measures | Coplan 2009 | (42) |
| Strange Stories task and Eyes Test | Theory of mind and social cognition | Inter-rater reliability reported in original task descriptions; internal consistency variable | Acceptable over short intervals in published work | Construct validity for advanced theory-of-mind reasoning | Clegg 2005 | (44, 45) |
| Vineland Adaptive Behavior Scales (VABS) | Adaptive functioning across communication, daily living, socialisation, and motor domains | High in published normative work | Strong in published normative work | Concurrent validity with intellectual and developmental status | Miranda 2023 | (46) |
| Social Responsiveness Scale (SRS) | Autistic-like social behaviour and social communication | Good in published validation samples | Acceptable in published validation samples | Convergent validity with ASD clinical diagnosis | Rodas 2017 | (47) |

**Note. Numerical references in this table refer to entries in the main manuscript bibliography. Internal consistency, test-retest reliability, and validity statistics summarised here are drawn from the cited validation studies and instrument manuals; where included studies reported sample-specific psychometric estimates, those are additionally referenced under the corresponding row.**
